# Elucidating the power of arginine restriction: taming type I interferon response in breast cancer via selective autophagy

**DOI:** 10.1186/s12964-024-01858-6

**Published:** 2024-10-08

**Authors:** Apsana Lamsal, Sonja Benedikte Andersen, Ida Johansson, Marie-Catherine Drigeard Desgarnier, Camilla Wolowczyk, Nikolai Engedal, Marina Vietri, Geir Bjørkøy, Miriam S. Giambelluca, Kristine Pettersen

**Affiliations:** 1 Department of Biomedical Laboratory Science, Faculty of Natural Sciences, Norwegian University of Science and Technology, Trondheim, Norway; 2 Centre of Molecular Inflammation Research, Department of Cancer Research and Molecular Medicine, Faculty of Medicine and Health Sciences, Norwegian University of Science and Technology, Trondheim, Norway; 3 Centre for Cancer Cell Reprogramming, Institute of Clinical Medicine, Faculty of Medicine, University of Oslo, Montebello, Oslo Norway; 4 Department of Molecular Cell Biology, Institute for Cancer Research, Oslo University Hospital, Montebello, Oslo Norway; 5 Institute for Cancer Research, Department of Tumor Biology, Oslo University Hospital, Montebello, Oslo Norway; 6 Department of Circulation and Medical Imaging, Faculty of Medicine and Health Sciences, Norwegian University of Science and Technology, Trondheim, Norway; 7 Department of Clinical Medicine, Faculty of Health Science, UiT- The Arctic University of Norway, Tromsø, Norway

## Abstract

**Background:**

Type I interferons (IFN-I) are potent alarm factors that initiate cancer cell elimination within tumors by the immune system. This critical immune response is often suppressed in aggressive tumors, thereby facilitating cancer immune escape and unfavorable patient outcome. The mechanisms underpinning IFN-I suppression in tumors are incompletely understood. Arginase-1 (ARG1)-expressing immune cells that infiltrate tumors can restrict arginine availability by ARG1-mediated arginine degradation. We hypothesized that arginine restriction suppresses the IFN-I response in tumors.

**Methods:**

Comprehensive, unbiased open approach omics analyses, various in vitro techniques, including microscopy, qPCR, immunoblotting, knock-down experiments, and flow cytometry were employed, as well as ex vivo analysis of tumor tissue from mice. Several functional bioassays were utilized to assess metabolic functions and autophagy activity in cancer cells.

**Results:**

Arginine restriction potently induced expression of selective autophagy receptors, enhanced bulk and selective autophagy and strongly suppressed the IFN-I response in cancer cells in an autophagy-dependent manner.

**Conclusion:**

Our study proposes a mechanism for how tumor-infiltrating immune cells can promote cancer immune escape by dampening the IFN-I response. We suggest ARG1 and autophagy as putative therapeutic targets to activate the IFN-I response in tumors.

**Supplementary Information:**

The online version contains supplementary material available at 10.1186/s12964-024-01858-6.

## Background

Aggressive and metastasis-prone tumors are frequently characterized by an immune-suppressive tumor microenvironment (TME) [1, 2]. The establishment of such a tumor-promoting TME depends on the ability of cancer cells to recruit infiltrating immune-suppressive cells and release anti-inflammatory cytokines [3]. Also, a stringent regulation of type I Interferon (IFN-I) signaling can be an important mechanism to avoid immune surveillance [4]. Dampening of this innate immune response is associated with poor responses to cancer therapy, and this has led to multiple attempts to reactivate it to improve patient outcome [5–7]. However, insufficient response to treatment and treatment-associated toxicity remain major challenges and there is still an urgent need for superior treatment options [8]. Understanding the mechanisms for IFN-I suppression in aggressive tumors is a prerequisite for the development of treatment strategies that can overcome this hurdle.

We recently showed that the IFN-I response is constitutively active in metastatic-prone breast cancer cells in culture but silenced in primary tumors formed after injecting these cells into the mammary fat pad of immunocompetent mice [9]. These findings suggests that the IFN-I response is dampened by conditions in the TME. Both during infections driven by DNA pathogens [10, 11] and in tumors [12], macroautophagy (herein referred to as autophagy) can suppress the innate immunity by diminishing the IFN-I response. Autophagy can be triggered by extrinsic conditions; the process is particularly sensitive to nutrient availability, and strongly induced in response to amino acid starvation [13]. Although limited blood supply combined with rapid growth and high metabolic activity may cause general limitations in nutrient supply within solid tumors, there is also a possibility that autophagy may be regulated through local reduction of specific amino acids [14]. Some infiltrating immune cells express amino acid hydrolyzing enzymes. Arginase 1 (ARG1) and indoleamine 2,3-dioxygenase (IDO), which hydrolyzes L-arginine and L-tryptophan, respectively, are the only amino acid-depleting enzymes known to be extensively elevated in cancer [15, 16]. Thus, local L-arginine or L-tryptophan depletion represent separate autophagy-inducing conditions that may occur in the TME.

Increased ARG1 levels and reduced arginine abundance in the TME are hallmarks of aggressive cancer development [17]. This is clearly illustrated in murine pancreatic ductal adenocarcinoma (PDAC) tumors where L-arginine is the most strongly depleted amino acid, with a barely detectable level in the interstitial fluid (extracellular tumor environment) [18]. We have recently shown that infiltration of ARG1-expressing immune cell and markedly increased arginine turnover in breast tumors [19] coincides with IFN-I suppression [9]. Based on these findings we hypothesized that limited arginine supply, due to ARG1-producing myeloid cells recruited to the tumor, would accelerate autophagy in transformed cancer cells and that this, in turn, would dampen the IFN-I response.

We found that arginine starvation served as a strong signal for the induction of specific autophagy receptors, especially SQSTM1, and boosted autophagic activity in breast cancer cells. Moreover, we found a selective turnover of mitochondria and recruitment of autophagic proteins to cyclic GMP–AMP synthase (cGAS) positive micronuclei. The enhanced autophagy in response to arginine restriction caused a striking reduction in cGAS-stimulator of interferon gene (cGAS-STING) signaling and attenuated a constitutive IFN-I response in the cancer cells. Our findings identify ARG1 from infiltrating immune cells and the autophagy process as potential therapeutic targets to reactivate the IFN-I response in tumors.

## Methods

### Cell lines and cell culture

66cl4 and MDAMB231 were obtained and cultivated as described in the supplementary methods.

### Chemicals

Torin1 (Tocris, UK, 4247), bafilomycin A1 (Santa Cruz Biotechnology, Texas, sc-201550 A), FCCP (Sigma Aldrich, Germany, C2920), oligomycin (Sigma Aldrich, O4876), antimycin (Sigma Aldrich, A8674), rotenone (Sigma Aldrich, R8875).

### Mice experiments

Eight- to twelve-week-old female BALB/cJ mice were obtained from Janvier Labs, France. The tumors were initiated and resected and processed as in [9], and described in detail in supplementary methods.

### Mass spectrometry analysis for tumor sections

Proteins from 66cl4 and 67NR mammary breast tumors from mice were isolated by homogenization in lysis buffer and analyzed by LC-MS/MS as described in [9], and in supplementary methods.

### Arginine starvation

Cells were seeded in 6 well culture plates and grown to 60–70% confluence. The cells were then given medium with various arginine concentrations for 24–48 h as described in the supplementary methods.

### Mass spectrometry analyses for arginine starved cells

Cells were grown in SILAC medium with various arginine concentrations (see “Arginine starvation”) for 24–48 h, then harvested and prepared for MS analyses as described earlier [9]. For detailed procedure for MS analysis see supplementary methods.

### Proteomics data analysis and bioinformatics analysis

MS data were processed to quantify proteins using MaxQuant v.2.0.3.0 [20]. The raw files were inspected using the open workflow provided in FragPipe [21] to determine optimal search criteria, and as in our previous analysis [9]. The proteomics and bioinformatics analyses are described in detail in supplementary methods.

### Quantitative real-time PCR

cDNA synthesis and RT-PCR was performed as described in the supplementary methods.

### Immunoblotting

Cells were harvested in 8 M urea lysis buffer (8 M urea, 0.5% (v/v) Triton X-100, 100 mM DTT, 1x Complete^®^ protease inhibitor (Roche, Switzerland) and 8x phosphatase inhibitor cocktail II and III (Sigma Aldrich). Protein concentration was measured at 595 nm using BioRad protein assay dye reagent (Bio-Rad, California, #500-0006) and the extracts were subjected to western blot as described in detail in supplementary methods. Uncropped images of all western blots are shown in Fig. S8.

### Immunofluorescence

MDAMB231 cells were treated and imaged as described in the supplementary methods.

### Antibodies

All antibodies used are listed in the supplementary methods.

### LDH sequestration assay for evaluation of bulk autophagy

LDH sequestration was determined as described previously [22, 23] with slight modifications and are described in detail in the supplementary methods.

### Metabolic analyses of glycolytic and mitochondrial function using seahorse XF96 analyzer

To evaluate metabolic function, oxygen consumption rate (OCR) and extracellular acidification rate (ECAR) were quantified using a Seahorse XF96 Analyzer (Agilent Technologies, California) as described in the supplementary methods.

### Analysis of mitochondrial membrane potential

66cl4 and MDAMB231 cells were treated, and mitochondrial membrane potential analyzed as explained in the supplementary methods.

### Generation of stable cell lines expressing inducible mito-mKeima or LDHB-mKeima

The monomeric Keima (mKeima) protein is a dual-emission fluorescent autophagy biosensor that emits light at 620 nm, while the wavelength for excitation depends on the pH [22, 24]. Here we used mKeima for evaluation of general (bulk) autophagic flux versus autophagic flux of mitochondria. Stable cell lines expressing LDHB-mKeima (cytosolic LDHB fused to mKeima; for measuring bulk autophagy) or mito-mKeima (the mitochondrial matrix-targeting presequence of COX VIII fused to mKeima; for measuring mitophagy) were generated by lentiviral transduction as in [22] and as described in the supplementary methods.

### Assessing autophagic flux by flow cytometry

MDAMB231-LDHB-mKeima and MDAMB231-mt-mKeima cells were treated, and autophagy and mitophagy flux, respectively, analyzed as described in the supplementary methods.

### Knocking down autophagy-associated genes by siRNAs

Expression of SQSTM1, TAX1BP1, ATG7 and ATG13 was knocked down in MDAMB231 cells using reverse transfection, as described in the supplementary methods.

### Code availability

The scripts for proteomics data analysis were extracted from respective R packages on CRAN and Bioconductor and can be requested.

### Statistical analyses

Statistics for MS analyses are described under the proteomics data analysis and bioinformatics analysis sections in the supplementary methods. All other statistical analyses were performed in GraphPad Prism 9. Values are expressed as mean ± standard error of the mean (SEM) if not otherwise stated. Details about statistical analyses are specified in the figure legends. “N” refers to the number of biologically independent experiments. p-value < 0.05 was considered statistically significant and is labeled with *, *p* < 0.01 is labeled with **, *p* < 0.001 is labeled with *** and *p* < 0.0001 is labeled with ****.

## Results

### Arginine restriction downregulated the IFN-I response in aggressive breast cancer cells

**Fig. 1:**
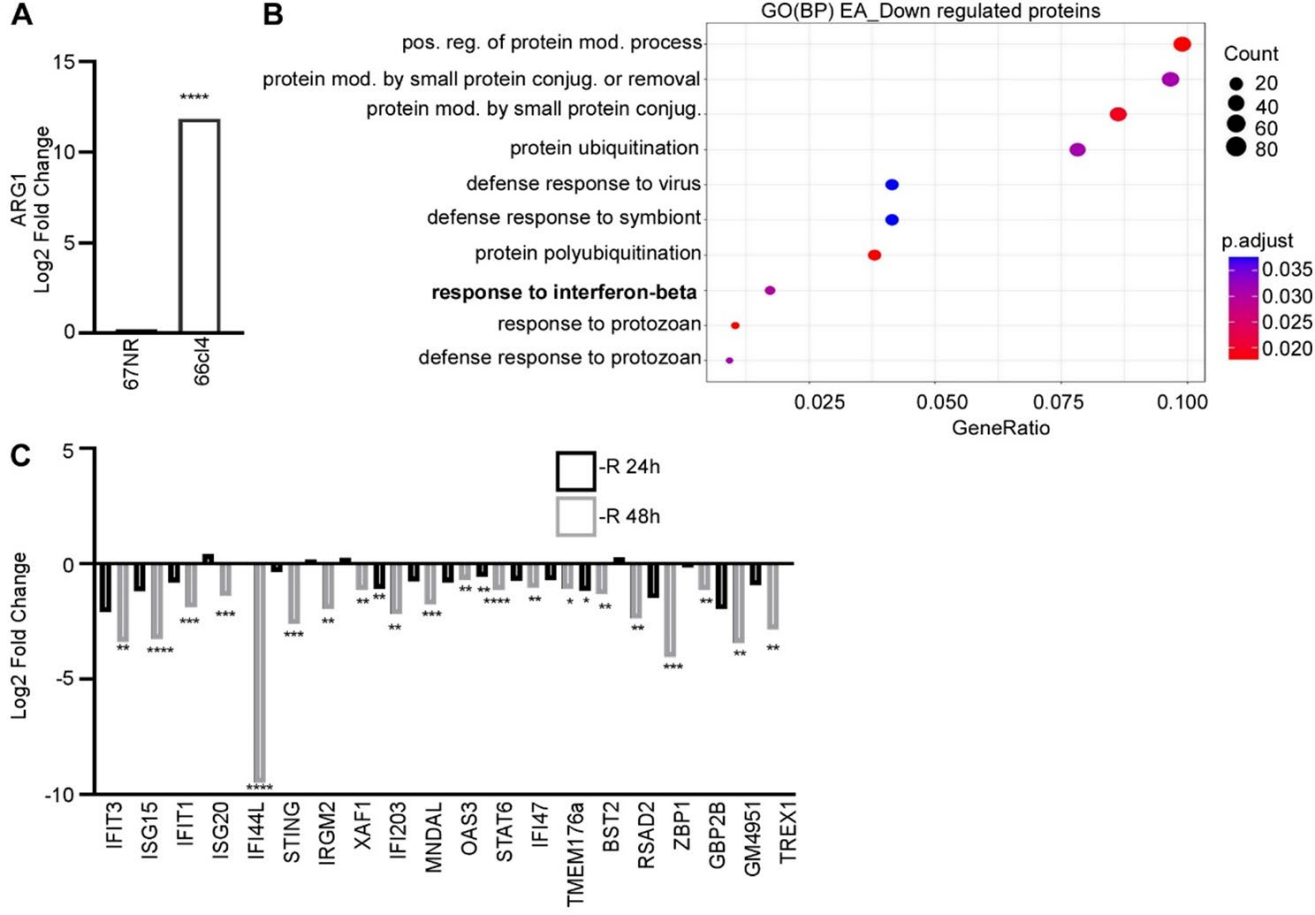
Low arginine abundance dampened IFN-I response in aggressive cancer cells. (**A**) Arginase 1 (ARG1) protein expression in 66cl4 tumors (*N* = 6) relative to 67NR tumors (*N* = 5) determined by mass spectrometry (MS) analysis. (**B**) 66cl4 cells were grown for 48 h in full medium (400 µM arginine) or without arginine, and protein extracts were analyzed by MS. Gene ontology (GO) functional enrichment analyses of biological processes (BP) for proteins with a reduced expression in arginine-starved cells (*N* = 6) relative to controls (*N* = 5). mod: modification, pos: positive, reg: regulation, neg: negative. (**C**) Downregulated IFN-I-related proteins, determined by MS analysis, in 66cl4 cells grown without arginine (-R) for 48 h (*N* = 6) or 24 h (*N* = 6) relative to full medium. For MS analysis, statistical significance was evaluated using student’s t-test corrected using Benjamini-Hochberg. *p < 0.05, ** *p* < 0.01, *** *p* < 0.001 and **** *p* < 0.0001

We hypothesized that ARG1 activity, and accordingly local arginine restriction, is a condition in the TME that can down-regulate the IFN-I response in tumors. We have already shown high ARG1 expression and reduced arginine abundance in IFN-I-suppressed 66cl4 tumors, as compared to IFN-I-expressing 67NR tumors [19]. After confirming these results by quantifying ARG1 protein expression by MS/MS in 66cl4 and 67NR tumors (Fig. 1A), we addressed whether arginine restriction limits the IFN-I response in 66cl4 cells. For this, we compared the proteome of 66cl4 cells grown in full, nutrient-rich medium or arginine-deficient medium for 48 h. Principal component analysis of the 4704 detected proteins showed a high degree of similarity between the biological replicates of each condition (Fig. S1A) and a clear difference in proteome composition between cells grown with or without arginine. Compared with controls, 66cl4 cells grown in arginine-free medium displayed 410 upregulated proteins (log2FC > 1, padj < 0.05) and 845 downregulated proteins (log2FC<-1, padj < 0.05) (Fig. S1B). To understand the biological processes (BP) linked to the differentially expressed proteins, we performed gene ontology (GO) enrichment analyses of the proteomes. Strikingly, identified by this unbiased approach, one of the processes most significantly reduced in arginine-starved cells was “response to interferon-beta” (Fig. 1B). In addition, “defense response to virus” was clearly reduced (Fig. 1B). Similar biological processes were also downregulated following partial arginine restriction (Fig. S1C). We further examined the proteins associated with the response to interferon beta and other interferon-related proteins selected based on literature [7]. After 24 h of arginine starvation, 3 IFN-I-related proteins were significantly downregulated, while 48 h arginine starvation downregulated a set of 20 proteins related to the IFN-I response (Fig. 1C). Cheng et al. [25] have previously performed RNA sequencing of arginine starved MDAMB231 cells, a human aggressive breast cancer cell line. When analyzing the RNA sequencing data from this earlier study, we found reduced expression of several IFN-I-related genes after 48 h arginine starvation (Fig. S1D). These results suggest that arginine restriction dampens the IFN-I response.

### Arginine restriction reduced the abundance of micronuclei in aggressive breast cancer cells

**Fig. 2:**
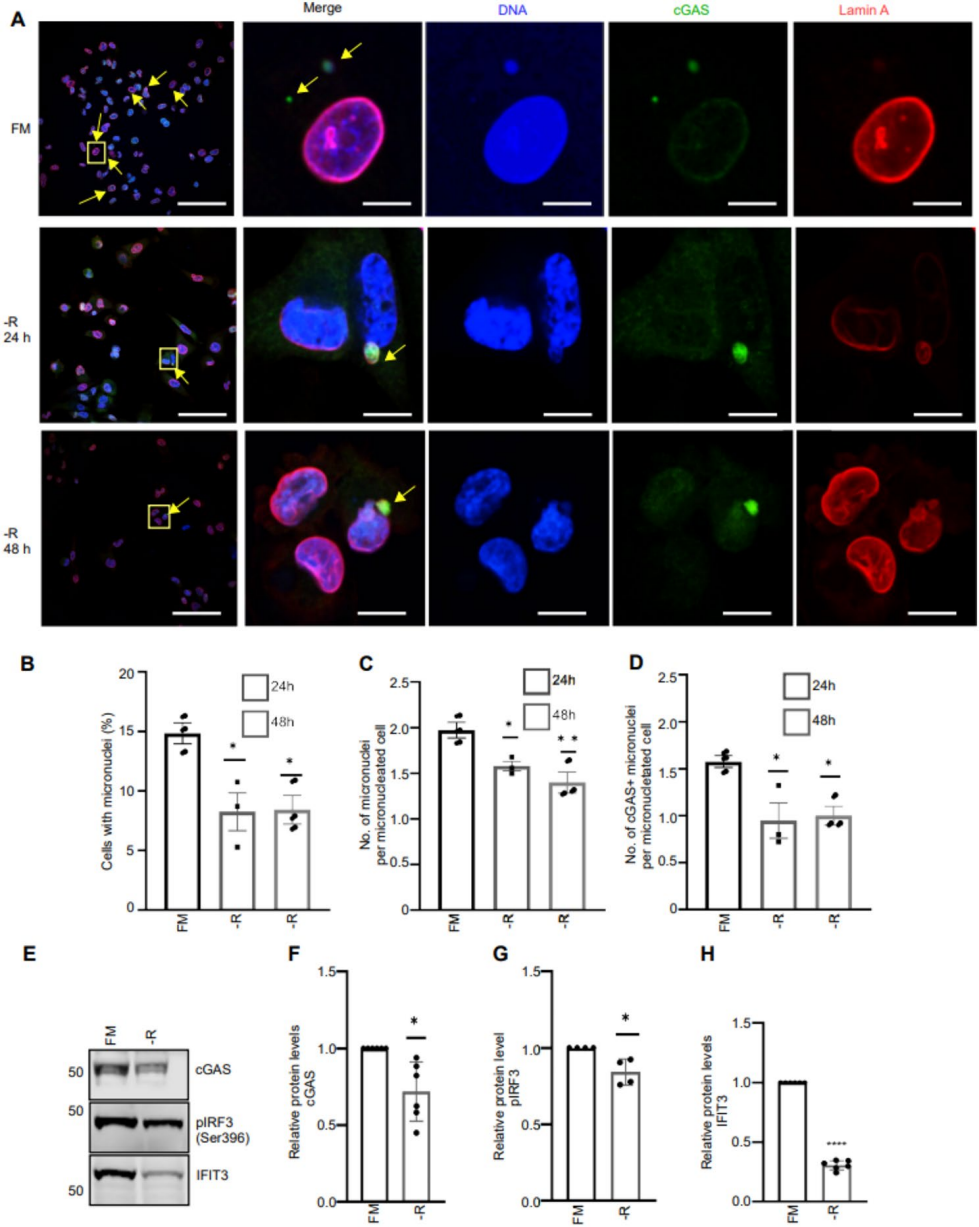
Arginine starvation reduced the abundance of micronuclei in aggressive breast cancer cells. (**A**) Representative immunofluorescence staining of micronuclei in MDAMB231 cells grown in full medium (FM) with 400 µM or no arginine (-R) for 24 h and 48 h. The cells were stained with cGAS (green), and Lamin A (red) antibodies and DNA was stained with Hoechst 33342 (blue). cGAS-positive micronuclei are highlighted by yellow arrows. Scale bar: 50 μm on the left and 5 μm on the selected cells within the yellow box. (**B**) Percentage of cells with micronuclei. (**C**) Number of micronuclei per micronucleated cell and (**D**) cGAS-positive micronuclei per micronucleated cell calculated from three independent experiments (*N* ≥ 300 cells per experiment). Bars represent mean ± SEM (*p < 0.05, **p < 0.01, one-way ANOVA, Dunnett’s multiple comparison). (**E**) Representative cGAS, pIRF3 and IFIT3 immunoblots of protein extracts from MDAMB231 cells grown for 48 h in arginine deficient medium relative to cells grown in full medium (400 µM arginine). (**F**-**H**) Quantification of cGAS (**F**), pIRF3 (**G**) and IFIT3 (**H**) protein levels. Total protein staining was used as loading control (Fig.S5). Bars represent mean ± SEM relative to full medium (*N* = 6 (cGAS and IFIT3), *N* = 4 (pIRF3), *p < 0.05, one sample t-test after log transformation)

Cytosolic DNA from micronuclei and mitochondria can activate the cGAS-STING pathway to induce the IFN-I response [26]. Recently, we have shown that micronuclei and in particular cGAS-positive micronuclei are highly abundant in aggressive human MDAMB231 cells [9], suggesting that DNA from ruptured micronuclei triggers the IFN-I response in these cancer cells in vitro. To determine whether arginine starvation affects the abundance of micronuclei, we subjected MDAMB231 cells to either full medium or arginine-free medium and examined micronucleated cells by confocal microscopy. Strikingly, arginine restriction significantly reduced both the frequency of cells containing micronuclei and the number of micronuclei per micronucleated cell (Fig. 2A-C).

cGAS has been reported to be recruited to membrane-ruptured micronuclei [27, 28]. We therefore examined if cGAS colocalized to micronuclei and examined the number of cGAS-positive micronuclei upon arginine starvation in MDAMB231 cells. As expected, the number of cGAS-positive micronuclei in the micronucleated cells were significantly reduced upon arginine starvation (Fig. 2A and D). The protein levels of cGAS, phosphorylation of IRF3 (pIRF3), an event downstream of cGAS-induced signaling [29], as well as the protein level of IFIT3 (a known IFN-I-induced protein [30]), were also reduced upon arginine starvation (Fig. 2E-H). Consistent with the reduction in micronuclei and cGAS-positive micronuclei in the aggressive human breast cancer cells, we found reduced cGAS levels also in 66cl4 cells upon arginine starvation (Fig. S2A-B). Together, these data suggest that micronuclei frequency and the cGAS-STING signaling axis is controlled by arginine availability.

### Arginine restriction elevated the expression of selective autophagy receptors and stimulated autophagic activity

**Fig. 3:**
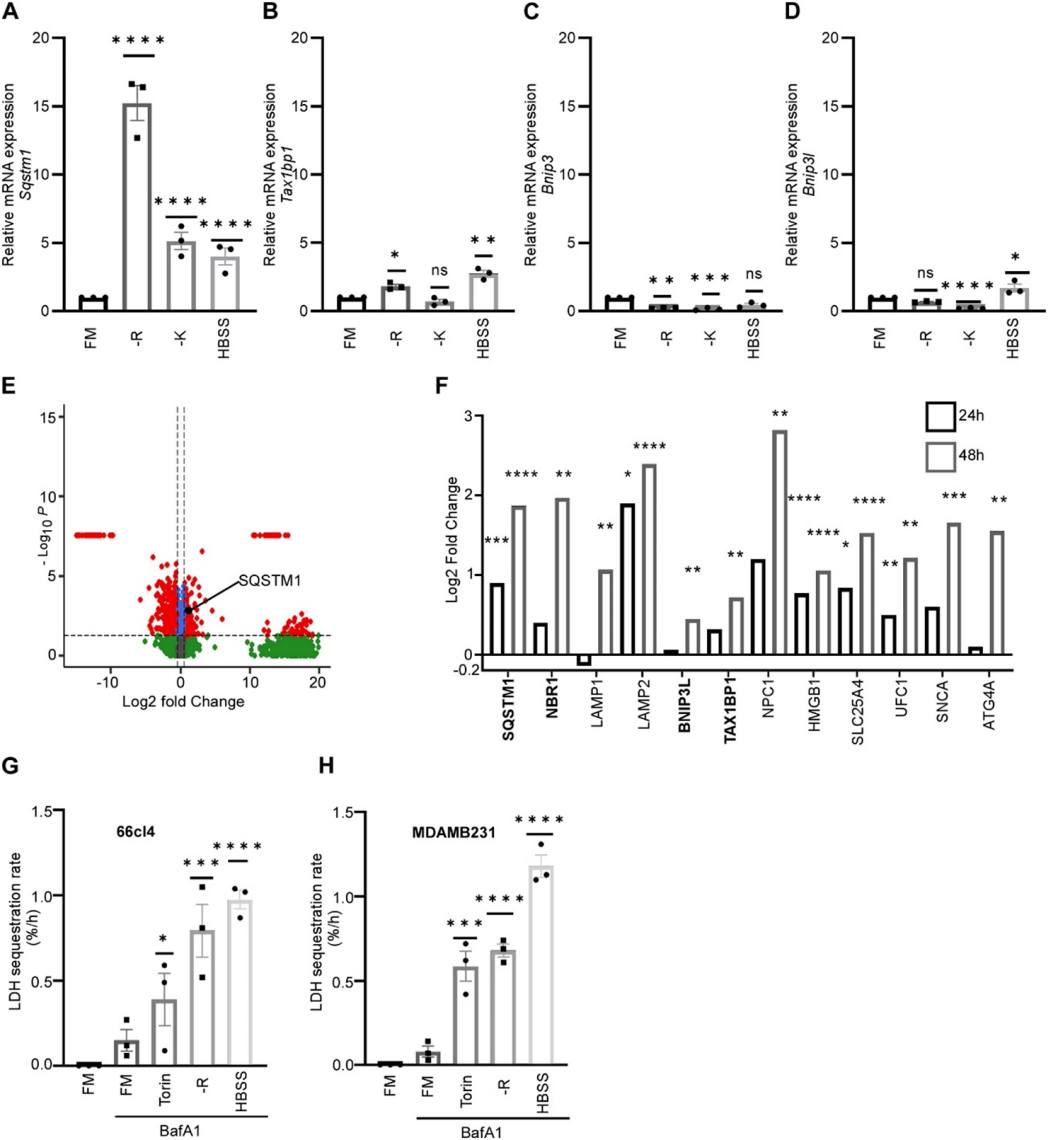
Arginine starvation induced selective autophagy receptors and stimulated autophagic flux. The mRNA level of the selective autophagy receptors *Sqstm1* (**A**), *Tax1bp1* (**B**), *Bnip3* (**C**) and *Bnip3l* (**D**) were analyzed in 66cl4 cells grown for 24 h in medium deficient of either arginine (-R), lysine (-K), or all amino acids (HBSS) as compared to full medium (FM). β-*Actin* was used as a housekeeping gene. Bars represent means ± SEM, one-way ANOVA, Dunnett’s multiple comparison test, after log transformation. (**E**) Volcano plot depicting differentially expressed proteins in 66cl4 cells grown in arginine deficient medium for 24 h relative to cells grown in full medium (400 µM arginine) (log2FC ± 0.5, *p* < 0.05). (**F**) Upregulated autophagy related proteins (autophagy receptors in bold) in 66cl4 cells grown in arginine deficient medium for 24 h (*N* = 6) and 48 h (*N* = 5) relative to full medium (400µM arginine) from MS analysis. Statistical significance was evaluated using student’s t-test corrected using Benjamini-Hochberg. (**G** and **H**) 66cl4 cells (**G**) or MDAMB231 cells (**H**) were grown either in full medium (FM), medium without arginine (-R) or in full amino acid starvation (HBSS) for 24 h, or in FM with Torin (overnight). The cells were treated with BafA1 (last 3 h) as indicated. LDH sequestration was determined at 21–24 h. Statistical significance was evaluated using repeated measures one-way ANOVA. For all bars: *p < 0.05, ** *p* < 0.01, *** *p* < 0.001 and **** *p* < 0.0001

Previous studies have uncovered that autophagy can counteract the IFN-I response in different settings ranging from senescence to neuro-inflammation [31]. However, in cancer cells, autophagy-mediated regulation of IFN-I response by factors present in the TME is poorly understood. To assess whether arginine starvation provoke the autophagy machinery in cancer cells, we analyzed the expression of several autophagy receptors following arginine starvation. Depriving 66cl4 cells of arginine significantly increased the transcript levels of the autophagy receptor *Sqstm1* (Fig. 3A). This response appeared to be arginine-specific, as starving the cells of either the essential amino acid lysine or all amino acids using Hank’s Balanced Salt Solution (HBSS), caused only about 1/3 of the increase in *Sqstm1* expression observed after arginine deprivation (Fig. 3A). Arginine restriction also significantly elevated the mRNA levels of another selective autophagy receptor; *Tax1bp1* (Fig. 3B), although the increase was more pronounced for *Sqstm1*. Other autophagy receptors such as *Bnip3* and *Bnip3l* were downregulated upon arginine starvation (Fig. 3C and D), indicating that arginine starvation does not cause a general induction of autophagy-receptors. To validate these transcriptomic changes at the protein level, we examined our MS-based proteomics data from arginine-starved 66cl4 cells. The proteome analysis showed that, among the autophagy receptors, only SQSTM1 was significantly upregulated at the protein level upon arginine starvation for 24 h (Fig. 3E and Fig. S2C). Prolonged incubation in arginine free medium (48 h) induced several other proteins involved in the autophagy process, including autophagy receptors (Fig. 3F). Consistently, a similar induction in SQSTM1 levels was evident in human breast cancer cells following arginine starvation (Fig. S2D).

We next asked whether arginine starvation increases autophagy activity. To monitor autophagic cargo flux in response to arginine restriction, we used the lactate dehydrogenase (LDH) sequestration assay [23, 32]. Under full-medium conditions LDH sequestration in 66cl4 cells was very low, indicating that the cells have a low basal autophagy activity (Fig. 3G). Strikingly, arginine starvation markedly enhanced LDH sequestration, producing an effect comparable to complete amino acid starvation using HBSS (Fig. 3G).

In line with the results from the mouse 66cl4 cells, we observed a similar induction in LDH sequestration rate in human breast cancer cells in response to arginine starvation (Fig. 3H). Together, these data suggest that arginine restriction both increases the transcript and protein levels of autophagy receptors and stimulates autophagic activity.

### cGAS positive micronuclei attracted the autophagic machinery upon arginine restriction

**Fig. 4:**
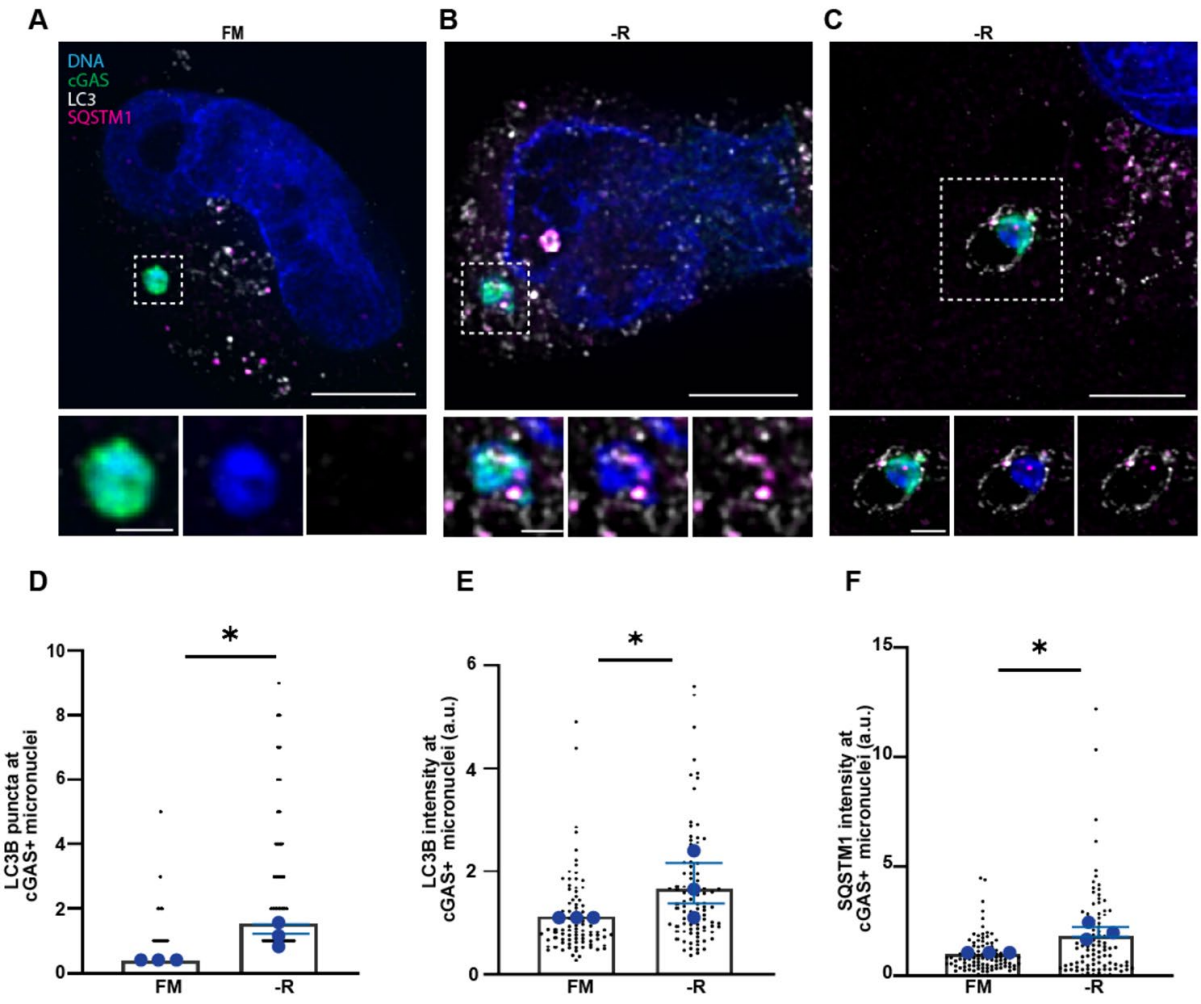
Micronuclei attracted autophagic machinery upon arginine restriction. Super resolution immunofluorescence staining of micronuclei in MDAMB231 cells grown in full medium (FM) with 400 µM (**A**) or no arginine (-R) for 24 h (**B** and **C**). The cells were stained with antibodies for cGAS (green), LC3B (grey) and SQSTM1 (magenta), and DNA was stained with Hoechst 33342 (blue). cGAS positive micronuclei are zoomed below the merged channels where merged channels, DNA/LC3B/SQSTM1 and finally only LC3B/SQSTM1 are shown, respectively. The image in (**B**) shows SQSTM1 and LC3B puncta around micronuclei and (**C**) shows micronuclei enclosed into LC3B positive puncta. Scale bar 5 μm for the overview panels and 1 μm for the zoomed panels. (**D**) Number of LC3B puncta at cGAS positive micronuclei. Black dots represent number of LC3 puncta and blue dots represent the relative fold change in each experiment from three independent experiments (*N* ≥ 30 cGAS positive micronuclei counted per experiment). Bars represent mean ± SEM (*p < 0.05, one sample t-test). (**E-F**) Normalized mean fluorescent intensity (MFI) (a.u.) of LC3B (**E**) and SQSTM1 (**F**) puncta around cGAS positive micronuclei calculated from three independent experiments (*N* ≥ 30 cGAS positive micronuclei counted per experiment). Black dots represent individual MFI of both LC3B and SQSTM1 puncta and blue dots represent the relative fold change in each experiment. Bars represent mean ± SEM (*p < 0.05, one sample t-test)

Micronuclei can associate with LC3 and SQSTM1 and are susceptible to autophagic degradation [33, 34]. Moreover, detection of micronuclei by the autophagy machinery requires membrane rupture and cytosolic exposure of DNA [35]. cGAS is a cytosolic DNA receptor that locates to ruptured micronuclei and may facilitate the autophagic process [35]. To investigate autophagic involvement around ruptured micronuclei upon arginine restriction, we performed super-resolution imaging of MDAMB231 cells co-immunostained for cGAS, LC3B and SQSTM1. We observed more LC3B positive puncta associated with cGAS positive (ruptured) micronuclei upon arginine restriction (Fig. 4A-D). These puncta where either in contact with or present inside the micronuclei (Fig. 4B and C) and displayed a higher mean fluorescent intensity of LC3B in arginine restricted cells compared to cells grown in full medium (Fig. 4E). SQSTM1 was also found in association with cGAS positive micronuclei, and arginine restriction caused an increased mean fluorescent intensity of SQSTM1 at these locations. (Fig. 4A-C, F). These findings suggest that arginine restriction stimulates recruitment of the autophagic markers SQSTM1 and LC3B to ruptured micronuclei, which could facilitate cGAS-mediated micronucleophagy.

### Arginine starvation reduced mitochondrial function and increased their turnover

**Fig. 5:**
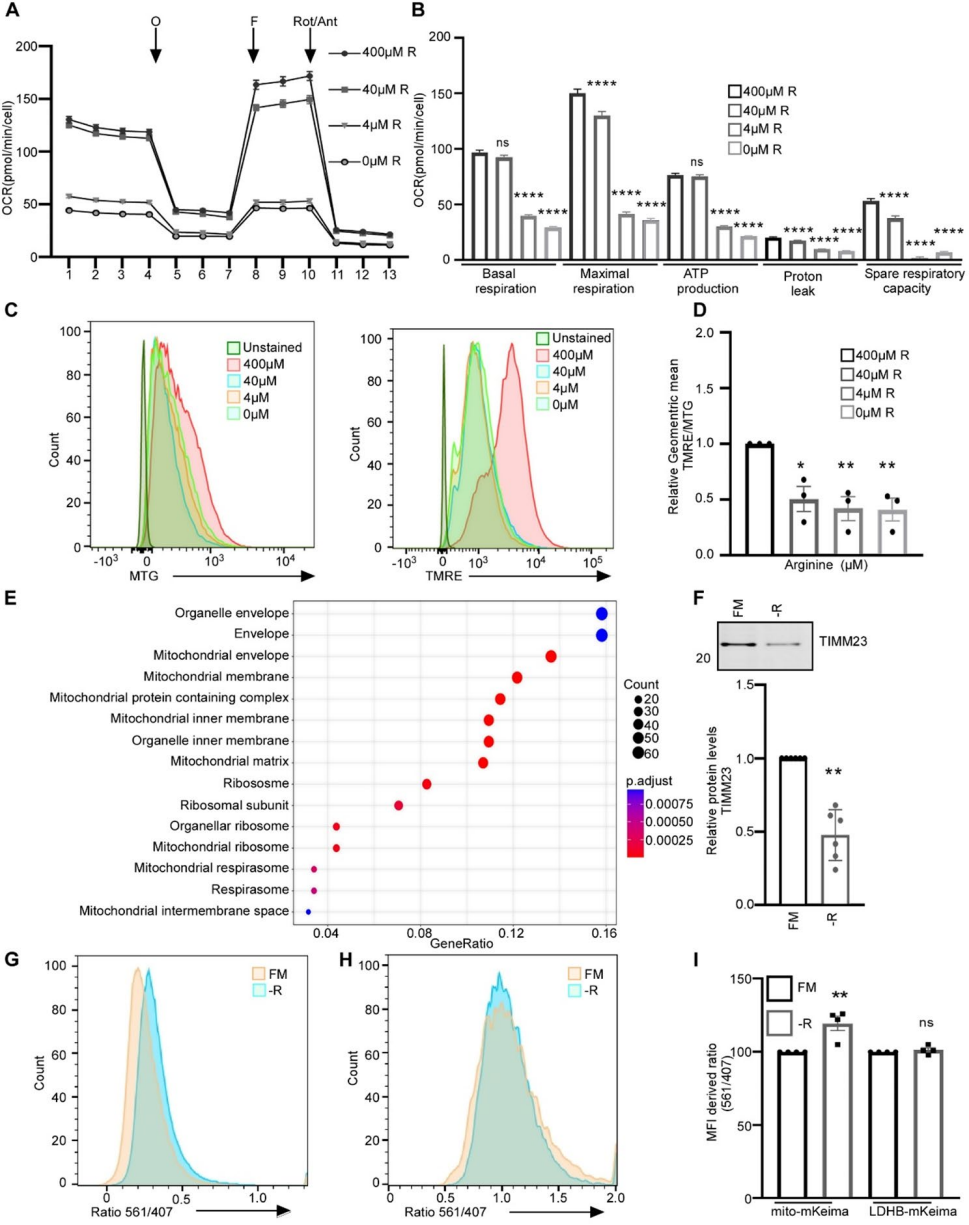
Arginine starvation caused impaired function and increased turnover of mitochondria. (**A**) Mito stress tests (Seahorse XF96 Analyzer) evaluating mitochondrial function of 66cl4 cells grown for 24 h in various arginine (R) concentration (*N* = 3, > 10 wells/condition). Oxygen consumption rate (OCR) before (basally) and after injections of oligomycin (O), FCCP (F) and rotenone/antimycin A (Rot/Ant) (mean ± SEM). (**B**) Basal OCR, proton leak, ATP production and spare respiratory capacity (SRC) (*N* = 3) (mean ± SEM, ANOVA, Dunnett’s multiple comparison test). Calculations based on (**A).** (**C**) Representative histograms from flow cytometry of 66cl4 cells grown in various arginine concentrations (24 h), stained with mitotracker green (MTG) or tetramethylrhodamine ethyl ester perchlorate (TMRE). (**D**) TMRE/MTG ratio in 66cl4 after growth in various arginine concentrations (24 h) (mean ± SEM, ANOVA, after log transformation, Dunnett’s multiple comparison test). (**E**) Gene ontology functional enrichment analyses of cellular components of proteins with reduced expression in 66cl4 cells grown in arginine deficient medium (*N* = 6) relative to full medium (400µM arginine, *N* = 5) (48 h). (**F**) Representative TIMM23 immunoblot and quantification (*N* = 6) from MDAMB231 cells grown in arginine deficient medium (-R) relative to full medium (FM, 400 µM arginine) (48 h) (mean ± SEM, one sample t-test after log transformation). Total protein staining used as loading control is shown in Fig. S5. (**G-H**) MDAMB231 cells expressing mito-mKeima **(G)** or LDHB-mKeima **(H)** grown with 400µM arginine (full medium, FM) or no arginine (-R) (48 h). Representative histograms of the signal ratio by excitation at 561 and 407 nm are shown. (**I**) Average median ratio for signals by excitation at 561 and 407 nm in MDAMB231 cells expressing mito-mKeima or LDHB-mKeima grown in 400 µM arginine (full medium, FM) or without arginine (-R) (48 h) (mean ± SEM, One way ANOVA, after log transformation, Dunnett’s multiple comparison test). Values for control cells were set to 100. For all bars: *p < 0.05, ** *p* < 0.01, *** *p* < 0.001 and **** *p* < 0.0001

Cytosolic DNA from both micronuclei and mitochondria can activate cGAS signaling and increase IFN-I expression [26]. In line with this, we have recently shown that cancer cells with constitutive IFN-I response have elevated levels of mitochondrial DNA (mtDNA) in the cytosol in addition to a higher frequency of ruptured micronuclei, compared to cancer cells with dampened IFN-I expression [9]. We hypothesized that arginine starvation can dampen the IFN-I response by activating an autophagic response that results in degradation of organelles that are prone to leak DNA into the cytosol. Having shown that autophagic components locate to ruptured micronuclei upon arginine restriction, we next examined the mitochondrial response to arginine restriction. Since arginine restriction previously has been shown to compromise mitochondrial functions in human breast cancer cells [25], we aimed to answer whether mitochondrial damage upon arginine restriction is accompanied by accelerated autophagy of mitochondria (mitophagy). First, we confirmed, using a Seahorse XF96 Analyzer that mitochondrial functions are significantly reduced in arginine restricted 66cl4 cells. Arginine starvation blunted the basal oxygen consumption rate, maximum respiratory capacity, proton leak, ATP production, and spare respiratory capacity (Fig. 5A and B). In addition, arginine restriction reduced the glycolytic capacity of the cells (Fig. S3A and B). To further examine the mitochondrial functions in response to arginine starvation, we performed flow cytometry analyses using fluorescent dyes to determine mitochondrial membrane potential (tetramethylrhodamine ethyl ester perchlorate; TMRE). The mitochondrial membrane potential decreased upon limited availability of arginine for 24 h, in a concentration-dependent manner (Fig. 5C). The decrease was also significant when adjusting for mitochondrial mass using Mitotracker green (MTG) in 66cl4 cells (Fig. 5D).

When performing GO analysis for cellular components (CC) of proteins induced in arginine-starved 66cl4 cells, we found that various proteins associated with the “mitochondrial envelope” and “mitochondrial membrane” were upregulated upon arginine starvation (Fig. 5E and Fig. S3E for 48 h and 24 h starvation, respectively). This may be a compensatory mechanism for compromised mitochondrial function.

In line with previous reports [36] and as seen in 66cl4 cells, downregulation of mitochondrial activity was also observed in MDAMB231 cells after arginine restriction (Fig. S3C and D).

Damaged and depolarized mitochondria can be sequestered into autophagosomes for subsequent lysosomal degradation [37]. In line with this, the level of the inner mitochondrial membrane protein TIMM23 was significantly reduced in response to arginine restriction in MDAMB231 cells (Fig. 5F). To assess whether arginine restriction increased mitophagy flux in these cells, we utilized a monomeric Keima (mKeima)-based assay that discriminates between autophagy of cytosolic content and the autophagic degradation of mitochondria. We quantified autophagic flux of cytosolic content and mitochondria by following the acidification of the fusion proteins lactate dehydrogenase B (LDHB)-mKeima and mitochondrial (mito)-mKeima, respectively, using flow cytometry [22]. Upon arginine restriction, we observed an increased shift in mito-mKeima to the acidic compartment compared to the LDHB-mKeima, demonstrating selectivity towards mitochondria over cytosol, and hence mitophagy (Fig. 5G-I). In summary, these findings demonstrate that arginine starvation causes both mitochondrial damage and increased mitophagy.

### Autophagy exerted negative control of the IFN-I response

**Fig. 6:**
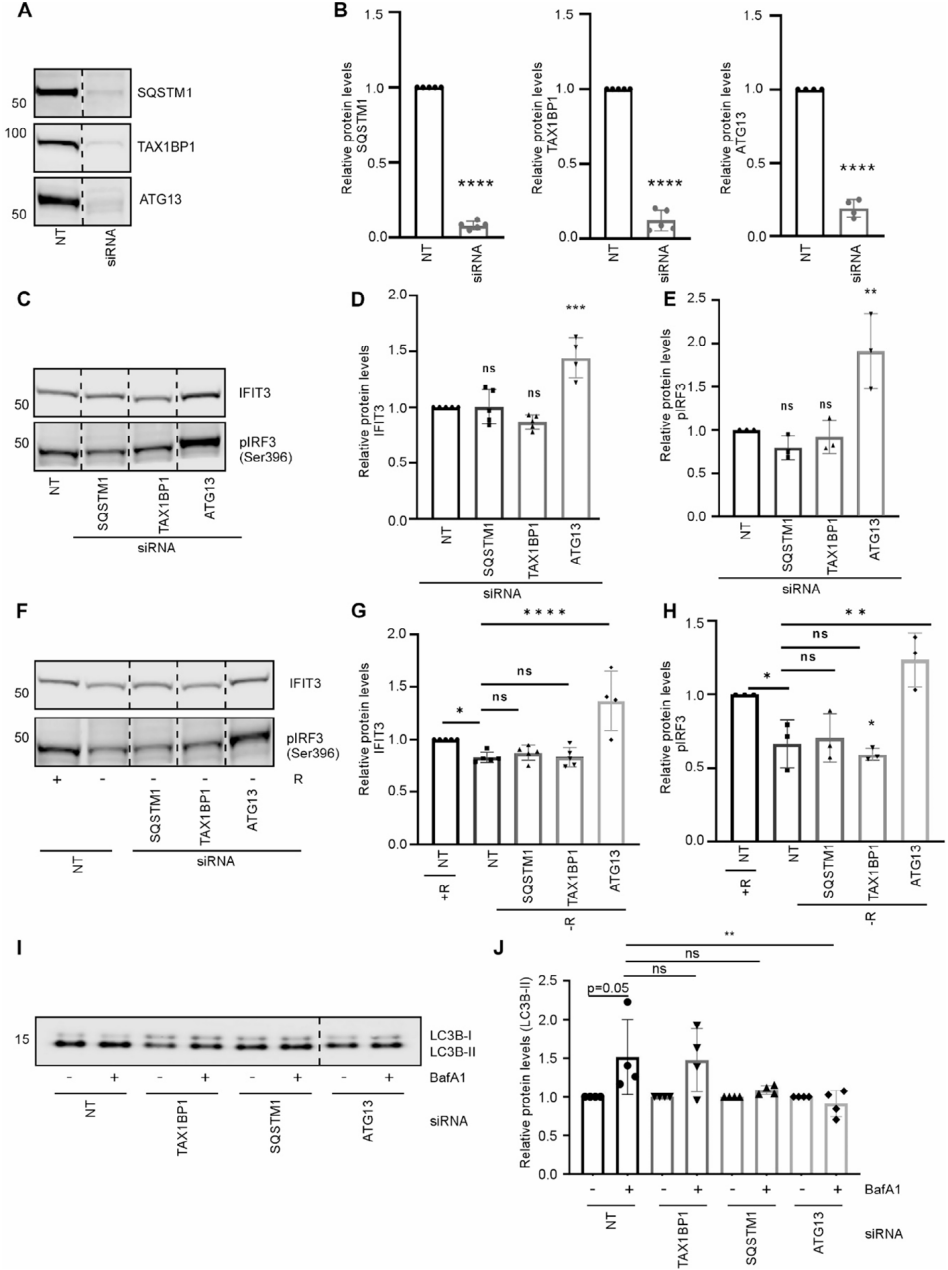
Autophagy negatively regulated the IFN-I response. (**A**) Representative immunoblots stained for SQSTM1, TAX1BP1 and ATG13 using protein extracts from MDAMB231 cells treated with siRNAs targeting SQSTM1, TAX1BP1 and ATG13, relative to non-targeting (NT) siRNA, demonstrating the efficiency of the various siRNAs. (**B**) Quantification of SQSTM1, TAX1BP1 and ATG13 protein level in siRNA treated MDAMB231 cells using total protein staining as loading control (Fig. S5). Bars represent mean ± SEM relative to NT siRNA (*N* ≥ 3, one sample t-test after log transformation). (**C**) Representative IFIT3 and pIRF3 immunoblots of protein extracts from MDAMB231 cells treated with siRNAs targeting SQSTM1, TAX1BP1 and ATG13, relative to NT siRNA. (**D**) Quantification of IFIT3 protein level in MDAMB231 cells after siRNA treatment, using total protein staining as loading control (Fig. S5). Bars represent mean ± SEM relative to NT siRNA (*N* ≥ 3, one sample t-test after log transformation). (**E**) Quantification of pIRF3 protein level in MDAMB231 cells after siRNA treatment, using total protein staining as loading control. Bars represent mean ± SEM relative to NT siRNA (*N* ≥ 3, one sample t-test after log transformation). (**F**) Representative IFIT3 and pIRF3 immunoblots of protein extracts from MDAMB231 cells treated with siRNAs targeting SQSTM1, TAX1BP1 and ATG13, relative to NT siRNA followed by cultivation with arginine (+ R; 400 µM arginine) or without arginine (-R) for 48 h post transfection. (**G**) Quantification of IFIT3 protein level in siRNA-treated MDAMB231 cells grown with (+ R) or without (-R) arginine for 48 h post transfection, using total protein staining as loading control (Fig. S5). Bars represent mean ± SEM relative to NT siRNA (*N* ≥ 3, one way ANOVA, Dunnett’s multiple comparison test, after log transformation). (**H**) Quantification of pIRF3 protein level, as described for IFIT3 in (**G**). **(I)** Representative LC3B immunoblot of protein extracts from MDAMB231 cells treated with NT siRNA or siRNAs targeting SQSTM1, TAX1BP1 and ATG13, with or without bafilomycin A1 (100 nM, 6 h) as indicated. **(J)** Quantification of LC3B-II protein level in MDAMB231 cells after siRNA and bafilomycin A1 treatment, using total protein staining as loading control (Fig. S5). Bars represent mean ± SEM for bafilomycin A treatment relative to control for each respective siRNA (*N* = 4, one way ANOVA, Tukey’s multiple comparison test, after log transformation). For all bars: *p < 0.05, ** *p* < 0.01, *** *p* < 0.001 and **** *p* < 0.0001. For all blots: Images originate from the same blot, but lanes are re-arranged as indicated by dotted lines

To examine whether elevated autophagy is important to downregulate cGAS-signaling and the IFN-I response, we performed siRNA-mediated knockdown of either *SQSTM1* or *TAX1BP1* (two of the autophagy receptors induced by arginine depletion), or *ATG13*, a key factor in the core autophagy machinery, in human MDAMB231 breast cancer cells. All siRNAs caused a significant reduction in their respective targets (Fig. 6A and B). While neither *SQSTM1* nor *TAX1BP1* knockdown altered the levels of IFIT3 and phosphorylated IRF3 protein, relative to control cells, the knockdown of *ATG13* caused a significant rise in both IFIT3 and pIRF3 (Fig. 6C-E). This indicates that the core autophagy machinery is important to control the IFN-I response under basal conditions. Upon arginine starvation, we found a reduction in pIRF3 and IFIT3 protein in control cells that was not counteracted in *SQSTM1* and *TAX1BP1* knock down cells (Fig. 6F-H). In contrast, *ATG13* knockdown interfered with the ability of arginine starvation to reduce the pIRF3 and IFIT3 levels. Although some reduction in the level of pIRF3 was observed in starved compared to non-starved *ATG13* knockdown cells (relative reduction ± SEM: 0.66 ± 0.03), the levels of both pIRF3 and IFIT3 were higher in *ATG13* knockdown cells compared to cells with functional autophagy (Fig. 6F-H). Differences in the ability of the siRNAs to interfere with pIRF3 and IFIT3 levels during arginine restriction may relate to differences in autophagy inhibition. To address this, LC3B-II accumulation in cells exposed to the various siRNA in combination with the lysosomal inhibitor bafilomycin A1, was used to report on autophagy activity. Of the three siRNAs, only ATG13 siRNA could significantly prevent LC3B-II accumulation in the cancer cells, indicating inhibited autophagy in ATG13 knock down cells (Fig. 6I and J). This is in line with the central role of ATG13 in the core autophagy process.

To further assess the importance of the core autophagy process in downregulating cGAS-signaling and the IFN-I response, we knocked down another core autophagy component, ATG7. As for knockdown of ATG13, ATG7 knockdown caused a significant rise in both IFIT3 and pIRF3 (Fig. S4A and B), underscoring that autophagy has a key role in IFN-I regulation. Knockdown of ATG7 also showed a clear tendency to counteract the starvation-induced reduction of pIRF3 and IFIT3 protein (Fig. S4C and D), although the effect was less prominent than during ATG13 knock down. The more potent ability of ATG13, compared to ATG7 knockdown, to counteract starvation-induced pIRF3 and IFIT3 reduction may relate to the rapid and strong reduction of ATG13 protein obtained, while reduction of ATG7 protein was more delayed (Fig. S4E and F). This delayed protein reduction was also reflected in the less prominent ability of ATG7 knockdown to inhibit autophagy (Fig. S4G and H). Together, these results indicate that dampening of the IFN-I response in arginine restricted cancer cells is dependent on the core autophagy machinery, but not on single autophagy receptors.

## Discussion

Innate immune responses play complex and dualistic roles in tumorigenesis, facilitating cancer cell elimination as well as contributing to immunological silencing and cancer immune escape, depending on the immunological components involved [38]. Aggressive tumors are associated with dampened IFN-I responses [4]. The absence of IFN-I signaling in tumors facilitates the escape of the cancer cells from the immune system [4]. Therefore, reactivation of this response is desirable as a therapeutic approach. Although the factors that initiate IFN-I signaling in response to pathogens are well understood, less is known regarding the signals that instigate negative control of innate immune pathways in cancer [39–41]. Our data support a model where tumor-infiltrating ARG1-positive immune cells restrict arginine availability in tumors and suppress the IFN-I response in cancer cells in an autophagy-dependent manner.

Most cancer cells, as well as many non-transformed cells, have a limited ability to produce arginine due to low expression of argininosuccinate synthase 1 (ASS1) [25, 42], thus making these cells vulnerable to arginine deficiency. This is the rationale behind the clinical cancer trials where arginine-depletion therapy is explored. However, arginine is also an important amino acid for T-cell function and immune destruction of malignant cells [17]. In accordance, arginine has been shown to enhance immune responses and to inhibit tumor growth in 4T1 breast tumor-bearing mice [43] and in B16-OVA melanoma-bearing mice [44]. In line with the important role of arginine in cancer immune destruction, conditions that restrict arginine availability, such as elevated levels of the arginine-depleting enzyme ARG1 [45] and suppressed expression of the arginine-generating enzyme ASS1 [36, 46, 47] is associated with poor prognosis in various malignancies. We find that arginine restriction dampens the IFN-I response in the cancer cells. This illustrates a novel mechanism for how arginine restriction can contribute to the formation of an immune suppressive TME. Our findings, alongside studies demonstrating how arginine-restriction can inhibit the activation of tumor-infiltrating lymphocytes [17], suggest that the metabolic disadvantage that arginine restriction may impose for the cancer cells, may be outweighed by the immune suppressive advantage induced by these conditions.

The role of autophagy in tumors is complex. While autophagy is important to prevent genotoxic damage that may facilitate cancer development, autophagy is an intrinsic cellular protection process, also in established cancer cells [48]. Although the mechanisms that fine-tune autophagy within tumors or normal tissues are not fully understood, it is established that the process is sensitive towards nutrient availability, with amino acid restriction being a strong autophagy-inducing condition. In line with others [49], we find that single amino acid restriction of arginine is sufficient to stimulate the autophagy process. Moreover, we find that a primary response to arginine restriction in cancer cells is a specific reprogramming of gene expression, including the rapid transcription of a defined set of autophagy receptors and other autophagy-related genes. Mining the RNAseq dataset from a recent study exploring transcriptional changes in the human breast cancer cell line MDAMB231 in response to arginine restriction also revealed elevated levels of the same autophagy receptors [25]. Notably, even though amino acid withdrawal typically is considered suppressive of overall protein synthesis, we also found that the protein level of these autophagy receptors was strongly increased. This underscores the importance of autophagy as a primary response when arginine levels decline.

There is an extensive crosstalk between autophagy and immune responses, with several studies demonstrating direct regulation of IFN-I signaling by key autophagy proteins [50–52]. In response to bacterial and viral infections, autophagy receptors SQSTM1, Nuclear dot protein 52 kDa, TAX1BP1, and Tripartite motif containing 23 can promote the degradation of retinoic acid-inducible gene I –like receptors, mitochondrial antiviral-signaling protein, and TIR-domain-containing adapter-inducing interferon-β, and thereby reduce IFN-I signaling [53–55]. In addition, cGAS and STING are also targeted by the autophagy machinery via SQSTM1 for subsequent degradation in the lysosome [56, 57]. cGAS can also act as an autophagy receptor and mediate clearance of micronuclei by directly interacting with LC3 in an LC3 interacting region-dependent manner [35]. However, most of these studies have been focused on responses to infections, and only recent studies have brought an attention to regulatory roles of autophagy in cancer innate immune response [12]. We find that reduced IFN-I response in arginine-starved cells coincides with increased bulk autophagy, mitophagy, and possibly micronucleophagy. Although a clear link between autophagy and DNA leakage has not been established, degradation of both mitochondria and micronuclei may decrease the abundance of DNA-leaking organelles, thereby hindering the exposure of DNA to the cytosol. In line with this, we find that cGAS-STING signaling is reduced upon arginine starvation in both murine and human breast cancer cells. Notably, the reduction in cGAS-STING signaling (and the IFN-I response) may also be a result of bulk autophagy sequestering and degrading cytosolic DNA, or the selective degradation of cGAS, STING and other IFN-I-response-related factors (such as that seen during infection). These options require further studies.

We observed elevated autophagy, reduced frequency of micronuclei and micronucleated cells, as well as suppressed IFN-I response in breast cancer cells subjected to arginine restriction. Also, in breast tumors with pronounced Arg1-mediated arginine turnover [19], the IFN-I response is suppressed [9]. In prostate cancer cells, however, arginine restriction has been shown to activate the cGAS-STING mediated IFN-I response [58]. This propose that cancer-specific responses to arginine restriction exist. Currently, both arginine depletion and supplementation have been considered possible treatment strategies in cancer. Unraveling the mechanisms underlying the cancer-specific responses would be essential to make informed treatment decisions.

Our results revealed that arginine restriction engaged a specific transcriptional response involving autophagy receptors, including *Tax1bp1* and *Sqstm1*. Nevertheless, we found that knock down of neither SQSTM1 nor TAX1BP1 could interfere with the IFN-I response under basal or arginine-restricted conditions, while ATG13 or ATG7 knock down clearly did. This may reflect functional redundancy between many of the selective autophagy receptors, while this is not the case for the core autophagy proteins ATG13 and ATG7. In line with the autophagy receptors functional redundancy, biallelic inactivating mutations in SQSTM1 in humans are not associated with excessive immune activation and autoimmunity [59]. Rather, these individuals develop childhood dementia [59]. This suggests that SQSTM1 is essential for suppressing neuronal degeneration, however, suppression of the IFN-I response may be compensated by other receptors. Which and how many other receptors that may take this compensatory role in this context is not known. However, multiple SQSTM1-like receptors, in addition to TAX1BP1, exist [60].

Our data suggest that autophagy can, in response to ARG1-mediated arginine restriction, negatively regulate IFN-I signaling in tumors. This is intriguing from a therapeutic perspective as it implies that ARG1 or autophagy can be targeted to reactivate IFN-I signaling. While many have tried to reactivate the IFN-I response in tumors using either interferons, STING agonists, or cyclic guanosine monophosphate–adenosine monophosphate-based nanoparticles, these approaches have generally been associated with considerable toxicity [8]. This underscores the need to find new druggable targets to activate the IFN-I response. Here, we identified two potential targets upstream of cGAS-STING signaling (ARG1 and autophagy) that can be inhibited using available pharmaceutical agents.

Several studies point to the anti-tumor effects of inhibiting ARG1. ARG1 inhibitors, such as *N*-hydroxy-nor-l-Arg and CB-1158, as well as ARG1-derived peptide vaccines, inhibit tumor growth in various murine models [61–64]. In line with the central role of myeloid cells in this response, myeloid-specific deletion of *Arg1* increased tumor CD8 + T cell infiltration and delayed formation of invasive disease [64]. Inhibition of ARG1 has also been shown to be associated with a proinflammatory TME, reduced myeloid cell-mediated immune evasion, increased T cell proliferation, and increased inflammatory cytokines [61, 64]. Additionally, inhibition of ARG1 using CB-1158 leads to increased IFN-I responsive transcripts in the tumor [61]. This is in line with our proposal, that ARG1 is a good therapeutic target to reactivate the IFN-I response. The effects of ARG1 inhibitors may also be further improved if combined with other anti-cancer agents. For instance, in the Lewis lung carcinoma mouse model, the ARG1 inhibitor (OAT-1746) showed significantly improved effect when combined with an immune checkpoint inhibitor (anti-PD-1) and STING agonist (DMXAA) compared to OAT-1746 given as a monotherapy [65]. An additive effect when combining ARG1 inhibitors with anti-PD1 immune cell blockade has also been observed in murine pancreatic cancer [64]. Notably, numerous other arginase inhibitors are approved by the Food and Drug Administration (FDA) [66]. Further, the autophagy inhibitors chloroquine and hydroxychloroquine (both accepted by the FDA) have shown benefits in cancer patients, including breast cancer [67]. These beneficial effects may relate to increased IFN-I signaling and anti-tumor immunity. In a murine mammary tumor model, blocking autophagy by FIP200-deletion led to increased expression of genes involved in IFN responses and other immune pathways [68]. This was also accompanied by enhanced CD8 + T cell infiltration. Moreover, interfering with autophagy by inhibition of Vacuolar protein sorting 34 (VPS34), either genetically or pharmacologically, increased CCL5 and CXCL10-mediated CD8 + T cell infiltration in murine melanoma and colorectal cancer [69]. An anti-tumor immune response was also observed in murine renal cancer as VPS34 inhibition increased cGAS/STING-mediated IFN-I response [70]. Interestingly, and as observed for ARG1 inhibitors, VPS34 inhibition may synergize with PD-1/PD-L1 blockade or STING agonists. Combinational treatment significantly decreased tumor growth and prolonged survival in tumor-bearing mice compared to single agent administration [69, 70]. It is therefore tempting to speculate that ARG1 inhibitors and/or autophagy inhibitors alone or in combination with conventional therapies or immunotherapy could achieve superior clinical efficacy in cancer patients with aggressive phenotypes. Clinical studies addressing these possibilities are urgently awaited.

## Conclusions

Increased arginase activity and concurrent arginine restriction are exacerbated in highly immune-suppressed tumors with a high infiltration of arginase-positive myeloid immune cells. This study unravels how cancer cells can benefit from an arginine-restricted environment and use this metabolic condition to dampen the IFN-I response and thereby facilitate cancer immune escape.

## Electronic supplementary material

Below is the link to the electronic supplementary material.

Additional file 1. Supplementary figures S1 to S8.

Additional file 2. Supplementary methods.

## Data Availability

The mass spectrometry proteomics data is deposited to the ProteomeXchange Consortium via the PRIDE [71] partner repository with the dataset identifier PXD037288. All other data in this article can be requested.

## Abbreviations

ARG1: Arginase-1
ASS1: Argininosuccinate synthase 1
ATG7: Autophagy related 7
ATG13: Autophagy related 13
ATG4A: Autophagy related 4A cysteine peptidase
BNIP3: BCL2 interacting protein 3
BNIP3L: BCL2 interacting protein 3 like
BP: Biological processes
BST2: Bone marrow stromal cell antigen 2
CC: Cellular components
cGAS: Cyclic GMP–AMP synthase
DMEM: Dulbecco’s Modified Eagle Medium
GBP2B: Guanylate binding protein 2b
GM4951: Predicted gene 4951
GO: Gene ontology
HMGB1: High mobility group box 1
IFI203: Interferon activated gene 203
IFI44L: Interferon induced protein 44 like
IFI47: Interferon gamma inducible protein 47
IFIT1: Interferon induced protein with tetratricopeptide repeats 1
IFIT3: Interferon induced protein with tetratricopeptide repeats 3
IFN: Interferon
IFN-I: Type I Interferons
IRF3: Interferon regulatory factor 3
IRGM2: Immunity-related GTPase family M member 2
ISG15: Interferon-stimulated gene 15
ISG20: Interferon-stimulated gene 20
LAMP1: Lysosomal-associated membrane protein 1
LAMP2: Lysosomal-associated membrane protein 2
LC3B: Light chain 3 beta
LDH: Lactate dehydrogenase
LDHB: Lactate dehydrogenase B
MAP1LC3B: Microtubule associated protein 1 light chain 3 beta
Mito: Mitochondrial
MNDAL: Myeloid nuclear differentiation antigen like
MTG: Mitotracker green
NBR1: Neighbor of BRCA1 gene 1
NPC1: NPC intracellular cholesterol transporter 1
OAS3: 2'-5'-oligoadenylate synthetase 3
OCR: Oxygen consumption rate
pIRF3: Phosphorylated interferon regulatory factor 3
RSAD2: Radical S-adenosyl methionine domain containing 2
RT: Room temperature
SLC25A4: Solute carrier family 25 member 4
SNCA: Synuclein Alpha
SQSTM1: Sequestosome 1
STAT6: Signal transducer and activator of transcription 6
STING: Stimulator of interferon gene
TAX1BP1: Tax1 binding protein 1
TIMM23: Translocase of inner mitochondrial membrane 23
TME: Tumor microenvironment
TMEM176a: Transmembrane protein 176A
TMRE: Tetramethyl rhodamine ethyl ester perchlorate
TREX1: Three prime repair exonuclease 1
UFC1: Ubiquitin-fold modifier conjugating enzyme 1
VPS34: Vacuolar protein sorting 34
XAF1: XIAP associated factor 1
ZBP1: Z-DNA binding protein 1
