# Supplementary material for: Elucidating the power of arginine restriction: taming type I interferon response in breast cancer via selective autophagy
Source: Cell Commun Signal. 2024 Oct 8;22:481. doi: 10.1186/s12964-024-01858-6 (PMC11462705; doi:10.1186/s12964-024-01858-6)
Supplement: Supplementary file 1 — Additional file 1. Supplementary figures S1 to S8. [file 12964_2024_1858_MOESM1_ESM.pdf]

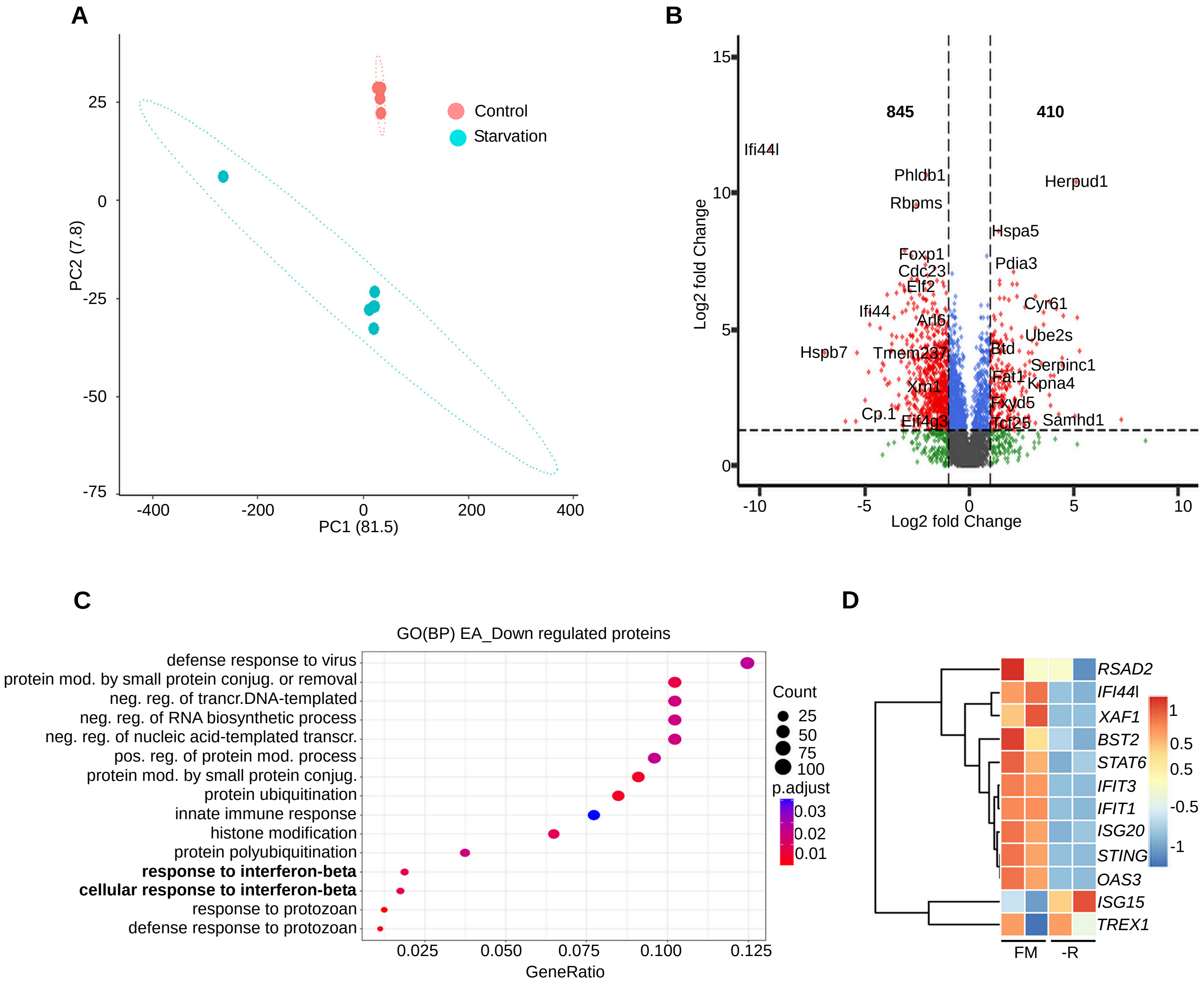
A
B
C
D
25
0
-25
-50
-75
PC2 (7.8)
-400
-200
0
200
400
PC1 (81.5)
Control
Starvation
15
10
5
0
Log2 fold Change
-10
-5
0
5
10
Log2 fold Change
845
410
Ifi44l
Phldb1
Rbpms
Herpud1
Hspa5
Foxp1
Cdc23
Elf2
Pdia3
Ifi44
Arl6
Cyr61
Ube2s
Hspb7
Tmem237
Btd
Serpinc1
Fat1
Kpna4
Xrn1
Fxyd5
Cp.1
Elf4g3
Tcf25
Samhd1
GO(BP) EA_Down regulated proteins
defense response to virus
protein mod. by small protein conjug. or removal
neg. reg. of trancr.DNA-templated
neg. reg. of RNA biosynthetic process
neg. reg. of nucleic acid-templated transcr.
pos. reg. of protein mod. process
protein mod. by small protein conjug.
protein ubiquitination
innate immune response
histone modification
protein polyubiquitination
response to interferon-beta
cellular response to interferon-beta
response to protozoan
defense response to protozoan
0.025
0.050
0.075
0.100
0.125
GeneRatio
Count
25
50
75
100
p.adjust
0.03
0.02
0.01
RSAD2
IFI44l
XAF1
BST2
STAT6
IFIT3
IFIT1
ISG20
STING
OAS3
ISG15
TREX1
1
0.5
0.5
-0.5
-1
FM
-R

Figure S1. Low arginine abundance dampened IFN-I response

(**A**) Principal Component Analysis of mass spectrometry data of proteins from 66cl4 cells grown in arginine deficient medium (N=6) or full medium (400 µM arginine) (N=5) for 48 h. (**B**) Volcano plot depicting differentially expressed proteins in 66cl4 cells grown for 48 h in arginine deficient medium or full medium (400 µM arginine) (log2FC±1.0, p<0.05). (**C**) GO (BP) functional enrichment analyses of proteins with a reduced expression in 66cl4 cells grown with limited access to arginine (4 µM arginine) for 48 h (N=6) relative to cells grown in full medium (400 µM) (N=5). conjug: conjugation, neg: negative, reg: regulation, transcr: transcribed, pos: positive, mod: modification, (**D**) Heatmap showing transcripts per million (after log10 transformation) of selected IFN-related genes expressed in MDAMB231 cells grown in arginine-deficient medium (-R) for 48 hours (N=2), relative to cells grown in full medium (FM) (N=2). The RNA expression data were sourced from Cheng et al 2018. The heatmap was generated using the pheatmap package (v1.0.12) in RStudio after log10 transformation of the expression values.

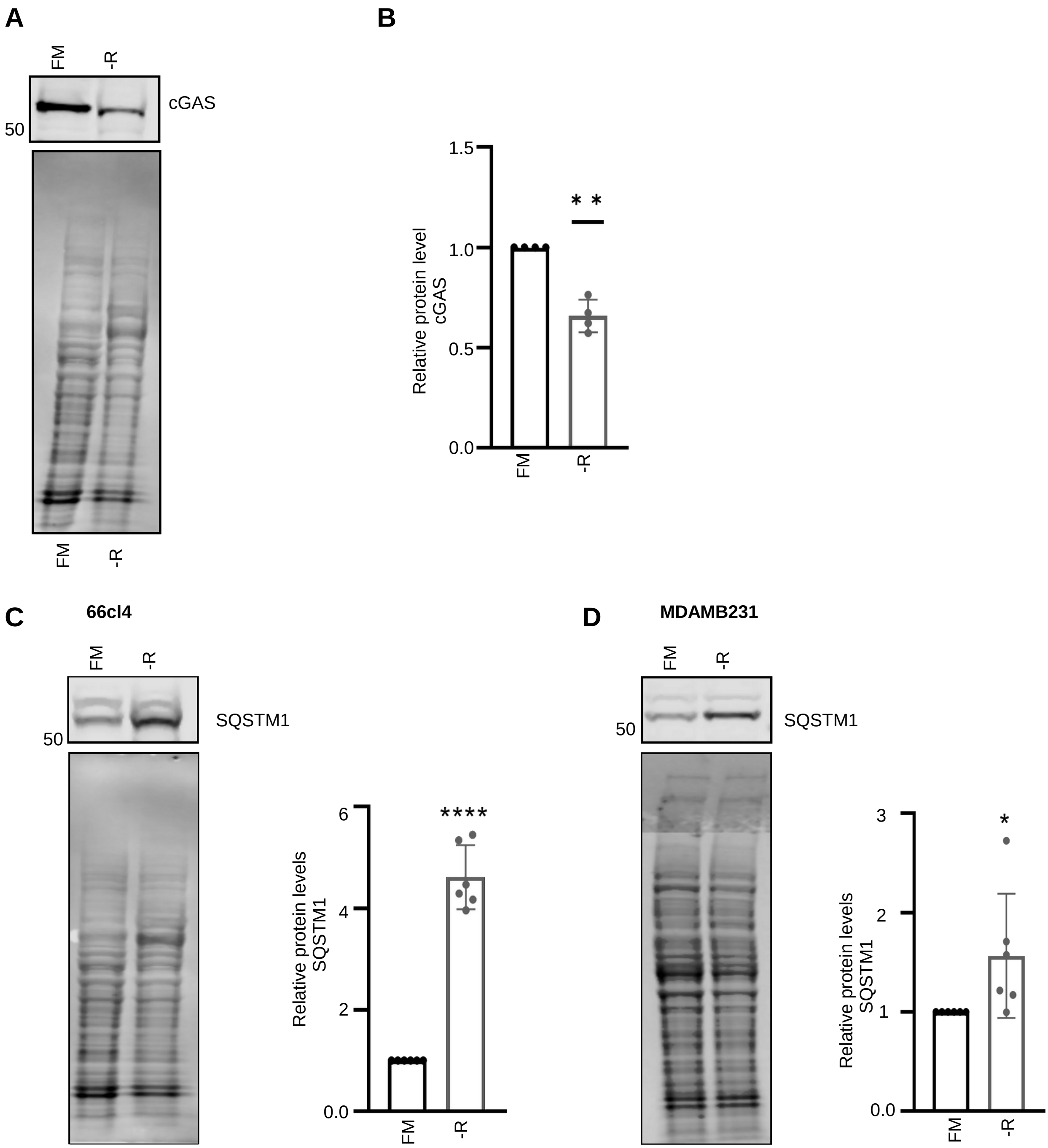
A
FM
-R
cGAS
50
FM
-R
B
1.5
1.0
0.5
0.0
* *
Relative protein level
cGAS
FM
-R
C
66cl4
FM
-R
SQSTM1
50
6
4
2
0.0
****
Relative protein levels
SQSTM1
FM
-R
D
MDAMB231
FM
-R
SQSTM1
50
3
2
1
0.0
*
Relative protein levels
SQSTM1
FM
-R

Figure S2. Arginine starvation reduced cGAS protein levels and induced SQSTM1 protein levels

(**A**) Representative cGAS immunoblot and corresponding total protein immunoblot of protein extracts from 66cl4 cells grown for 48 h in arginine deficient medium relative to cells grown in full medium (400 µM arginine). (**B**) Quantification of cGAS protein level was done using total protein staining shown in A as loading control. Bars represent mean ± SEM relative to full medium (N=4), **$p<0.01$, one sample t-test after log transformation. (**C**) Representative SQSTM1 and corresponding total protein immunoblot of protein extracts from 66cl4 cells grown for 24 h in arginine deficient medium relative to the cells grown in full medium (400 µM arginine). (**D**) Representative SQSTM1 and corresponding total protein immunoblot of protein extracts from MDAMB231 cells grown for 48 h in arginine deficient medium relative to the cells grown in full medium (400 µM arginine). Quantification of SQSTM1 protein level was done using total protein staining as loading control. Bars in C and D represent mean ± SEM relative to full medium (N=6), *$p<0.05$ and ****$p<0.0001$, one sample t-test after log transformation.

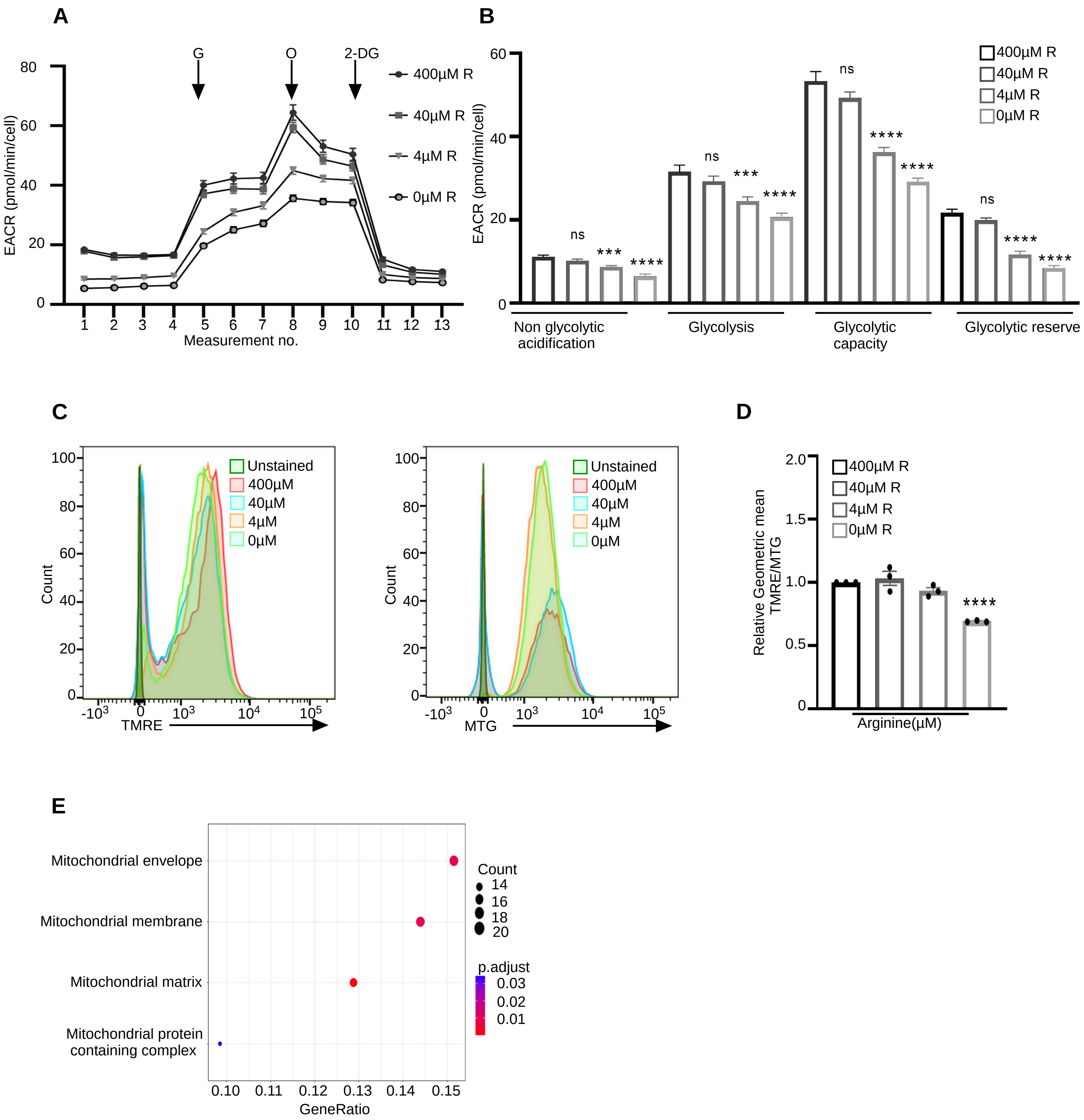
A
G
O
2-DG
EACR (pmol/min/cell)
Measurement no.
400μM R
40μM R
4μM R
0μM R
B
EACR (pmol/min/cell)
ns
***
****
Non glycolytic acidification
Glycolysis
Glycolytic capacity
Glycolytic reserve
C
Unstained
400μM
40μM
4μM
0μM
Count
TMRE
MTG
D
Relative Geometric mean TMRE/MTG
Arginine(μM)
E
Mitochondrial envelope
Mitochondrial membrane
Mitochondrial matrix
Mitochondrial protein containing complex
GeneRatio
Count
p.adjust

Figure S3. **Arginine starvation impaired glycolytic function, in addition to the impaired mitochondrial function**

(**A**) Glycolysis stress tests were performed on a Seahorse XF96 Analyzer to evaluate glycolytic function of 66cl4 cells grown for 24 h in either full medium (400µM arginine) or medium with various arginine (R) concentration (40 µM, 4 µM, 0 µM) (N=3, with a minimum of 11 wells per condition). The graph shows the extracellular acidification rate (ECAR) both before (basally) and after injections of glucose (G), oligomycin (O) and 2-deoxy-glucose (2-DG). (**B**) Data obtained from the experiments described in (**A**) was used to calculate non-glycolytic acidification, glycolysis, glycolytic capacity, and glycolytic reserve (N=3). Data is shown as mean ± SEM (ANOVA, Dunnett's multiple comparison test). (**C**) Representative histograms of MDAMB231 cells grown for 48 h in medium with various arginine concentration, stained with mitotracker green (MTG) (to determine mitochondrial mass) or tetramethylrhodamine ethyl ester perchlorate (TMRE) (mitochondrial membrane potential) and analyzed by flow cytometry. (**D**) Quantitation of TMRE/MTG ratio in MDAMB231 after growth for 48 h in various arginine concentrations. Bars represent mean ± SEM (ANOVA, after log transformation, Dunnett's multiple comparison test). (**E**) Gene ontology functional enrichment analyses of cellular components of proteins with a reduced expression in 66cl4 cells grown in arginine deficient medium for 24 h (N=6) relative to cells grown in full medium (400 µM arginine) (N=6). For all bars: *p <0.05, ** p <0.01, *** p <0.001 and **** p <0.0001.

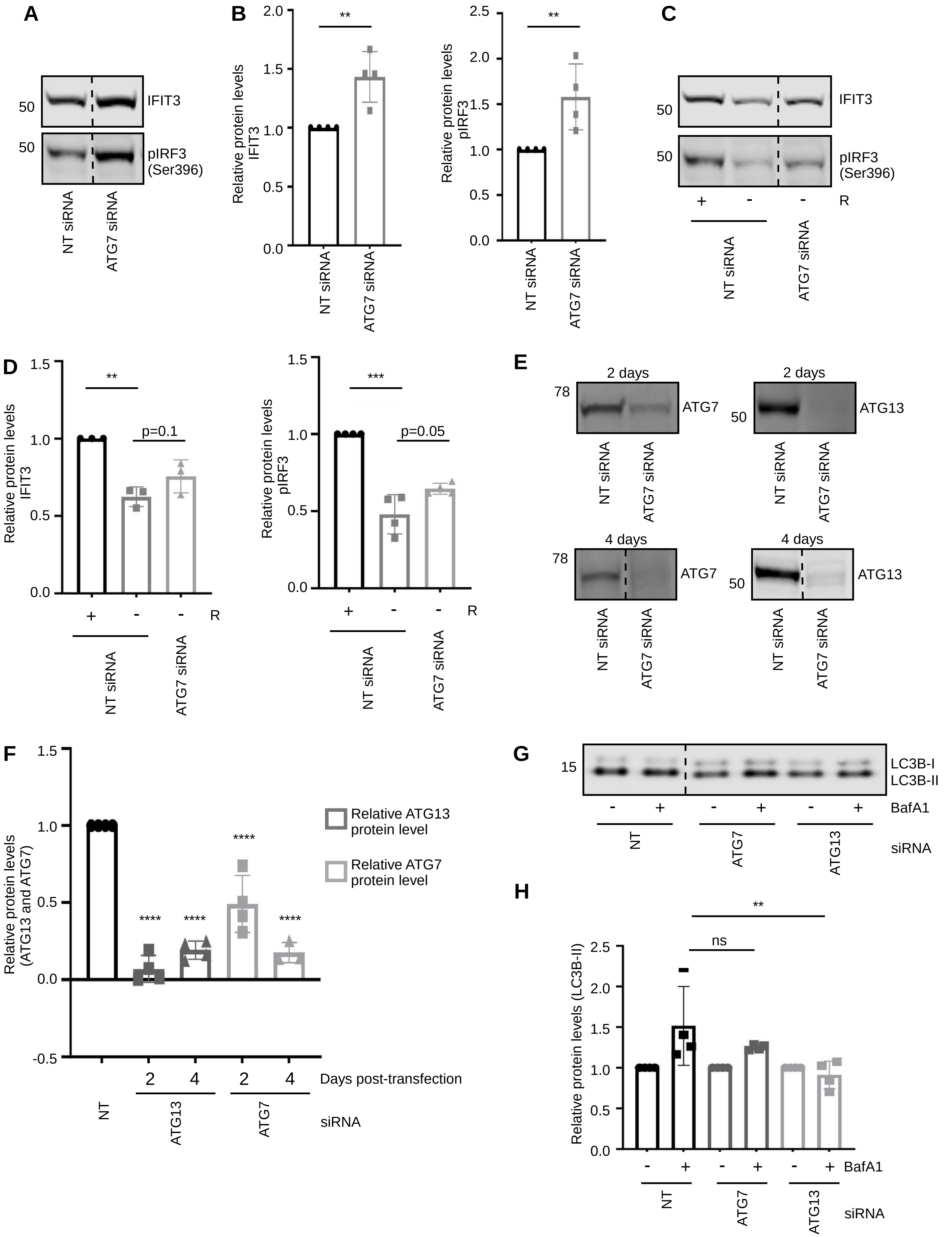
A
50
IFIT3
50
pIRF3
(Ser396)
NT siRNA
ATG7 siRNA
B
Relative protein levels
IFIT3
**
NT siRNA
ATG7 siRNA
Relative protein levels
pIRF3
**
NT siRNA
ATG7 siRNA
C
50
IFIT3
50
pIRF3
(Ser396)
+
-
-
R
NT siRNA
ATG7 siRNA
D
Relative protein levels
IFIT3
**
p=0.1
+
-
-
R
NT siRNA
ATG7 siRNA
Relative protein levels
pIRF3
***
p=0.05
+
-
-
R
NT siRNA
ATG7 siRNA
E
2 days
78
ATG7
NT siRNA
ATG7 siRNA
2 days
50
ATG13
NT siRNA
ATG7 siRNA
4 days
78
ATG7
NT siRNA
ATG7 siRNA
4 days
50
ATG13
NT siRNA
ATG7 siRNA
F
Relative protein levels
(ATG13 and ATG7)
Relative ATG13
protein level
Relative ATG7
protein level
****
****
****
****
2
4
2
4
Days post-transfection
NT
ATG13
ATG7
siRNA
G
15
LC3B-I
LC3B-II
-
+
-
+
-
+
BafA1
NT
ATG7
ATG13
siRNA
H
**
ns
Relative protein levels (LC3B-II)
-
+
-
+
-
+
BafA1
NT
ATG7
ATG13
siRNA

Figure S4. **Autophagy suppressed the IFN-I response**

(**A**) Representative IFIT3 and pIRF3 immunoblots of protein extracts from MDAMB231 cells treated with siRNAs targeting ATG7, relative to NT siRNA. **(B)** Quantification of IFIT3 and pIRF3 protein level in MDAMB231 cells after siRNA treatment, using total protein staining as loading control (Fig. S5). Bars represent mean ± SEM relative to NT siRNA (N=4, one sample t-test after log transformation). **(C)** Representative IFIT3 and pIRF3 immunoblots of protein extracts from MDAMB231 cells treated with siRNAs targeting ATG7, relative to NT siRNA followed by cultivation with arginine (+R; 400 µM arginine) or without arginine (-R) for 48 h post transfection. **(D)** Quantification of IFIT3 and pIRF3 protein level in siRNA-treated MDAMB231 cells grown with (+R) or without (-R) arginine for 48 h post transfection, using total protein staining as loading control (Fig. S5). Bars represent mean ± SEM relative to NT siRNA (N=4, one way ANOVA, Tukey's multiple comparison test, after log transformation). **(E)** Representative immunoblots stained for ATG7 or ATG13, as indicated, using protein extracts from MDAMB231 cells treated with siRNAs targeting ATG7 or ATG13, relative to non-targeting (NT) siRNA, demonstrating the efficiency of the various siRNAs. 2 and 4 days indicate days after transfection. **(F)** Quantification of ATG7 and ATG13 protein level in siRNA treated MDAMB231 cells using total protein staining as loading control (Fig. S5). Bars represent mean ± SEM relative to NT siRNA (N=4, one way ANOVA, Dunnett's multiple comparison test). **(G)** Representative LC3B immunoblot of protein extracts from MDAMB231 cells treated with NT siRNA or siRNAs targeting ATG7 and ATG13, with or without bafilomycin A1 (100nM, 6h) as indicated. **(H)** Quantification of LC3B-II protein level in MDAMB231 cells after siRNA and bafilomycin A1 treatment, using total protein staining as loading control (Fig. S5). Bars represent mean ± SEM for bafilomycin A treatment relative to control for each respective siRNA (N=4, one way ANOVA, Tukey's multiple comparison test, after log transformation). For all bars: *p <0.05, ** p <0.01, *** p <0.001 and **** p <0.0001, ns=not significant. For all blots: Images originate from the same blot, but lanes are re-arranged as indicated by dotted lines.

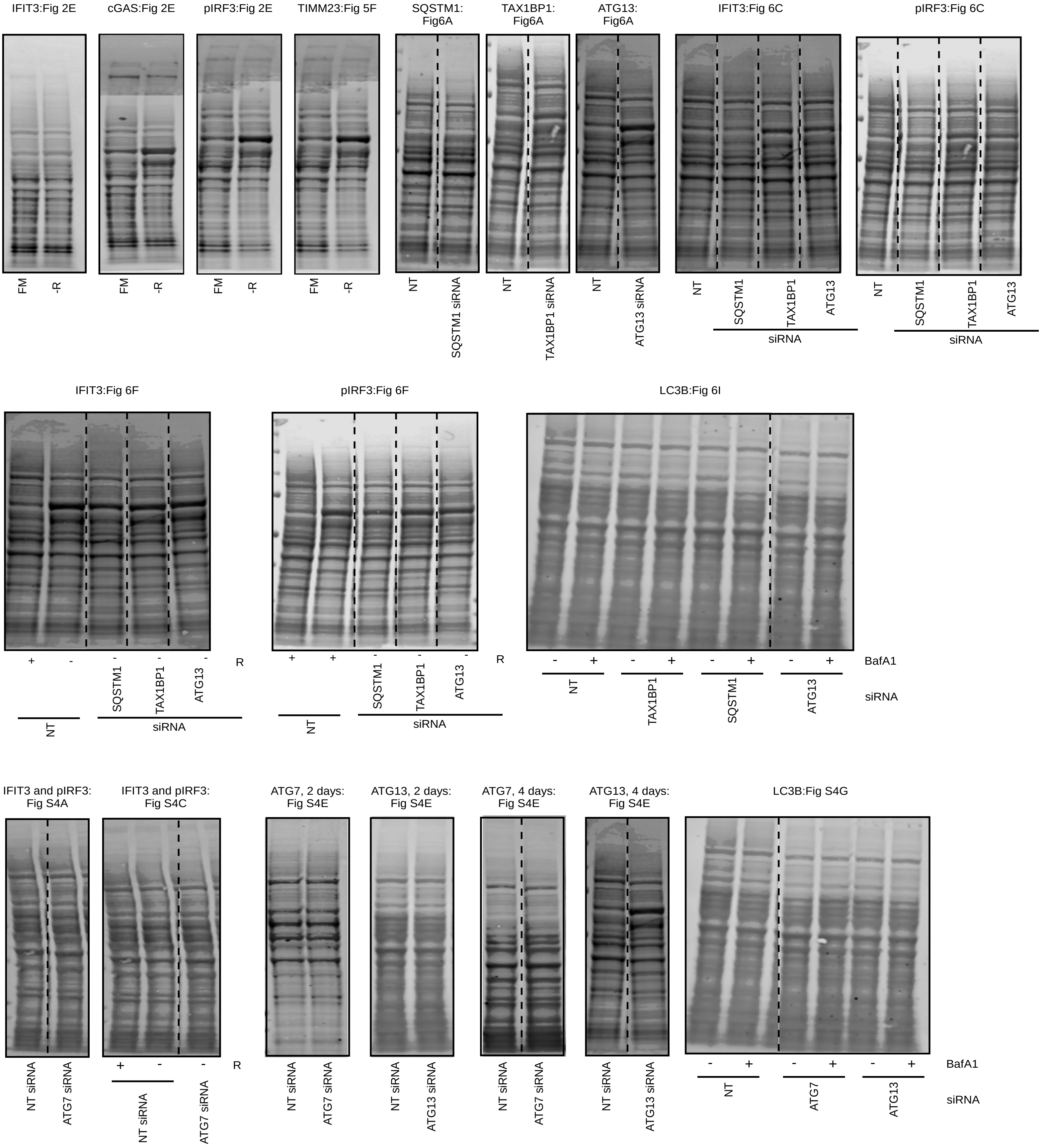
IFIT3:Fig 2E
cGAS:Fig 2E
pIRF3:Fig 2E
TIMM23:Fig 5F
SQSTM1: Fig6A
TAX1BP1: Fig6A
ATG13: Fig6A
IFIT3:Fig 6C
pIRF3:Fig 6C
FM
-R
NT
SQSTM1 siRNA
TAX1BP1 siRNA
ATG13 siRNA
SQSTM1
TAX1BP1
ATG13
siRNA
IFIT3:Fig 6F
pIRF3:Fig 6F
LC3B:Fig 6I
+
-
R
BafA1
IFIT3 and pIRF3: Fig S4A
IFIT3 and pIRF3: Fig S4C
ATG7, 2 days: Fig S4E
ATG13, 2 days: Fig S4E
ATG7, 4 days: Fig S4E
ATG13, 4 days: Fig S4E
LC3B:Fig S4G
NT siRNA
ATG7 siRNA
ATG13 siRNA
ATG7

Figure S5. Representative total protein stain of the respective immunoblots included.

Total protein staining was used as loading control in all the blots and the lanes shown were used to quantify and normalize the respective blots. Images related to the same main figure originate from the same blot, but lanes are re-arranged as indicated by dotted lines.

66cl4 TMRE/MTG gating strategy

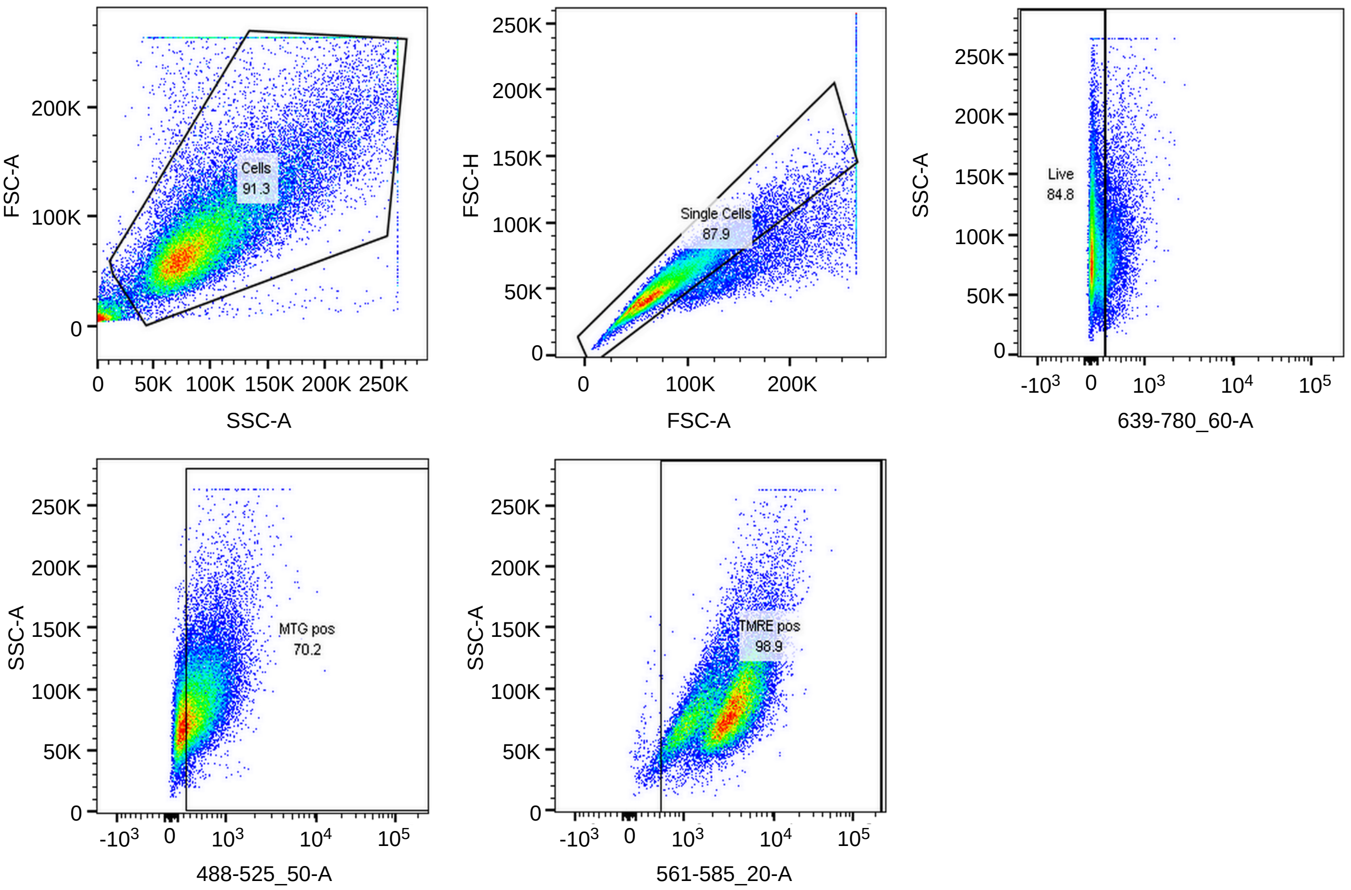

MDAMB231 TMRE/MTG gating strategy

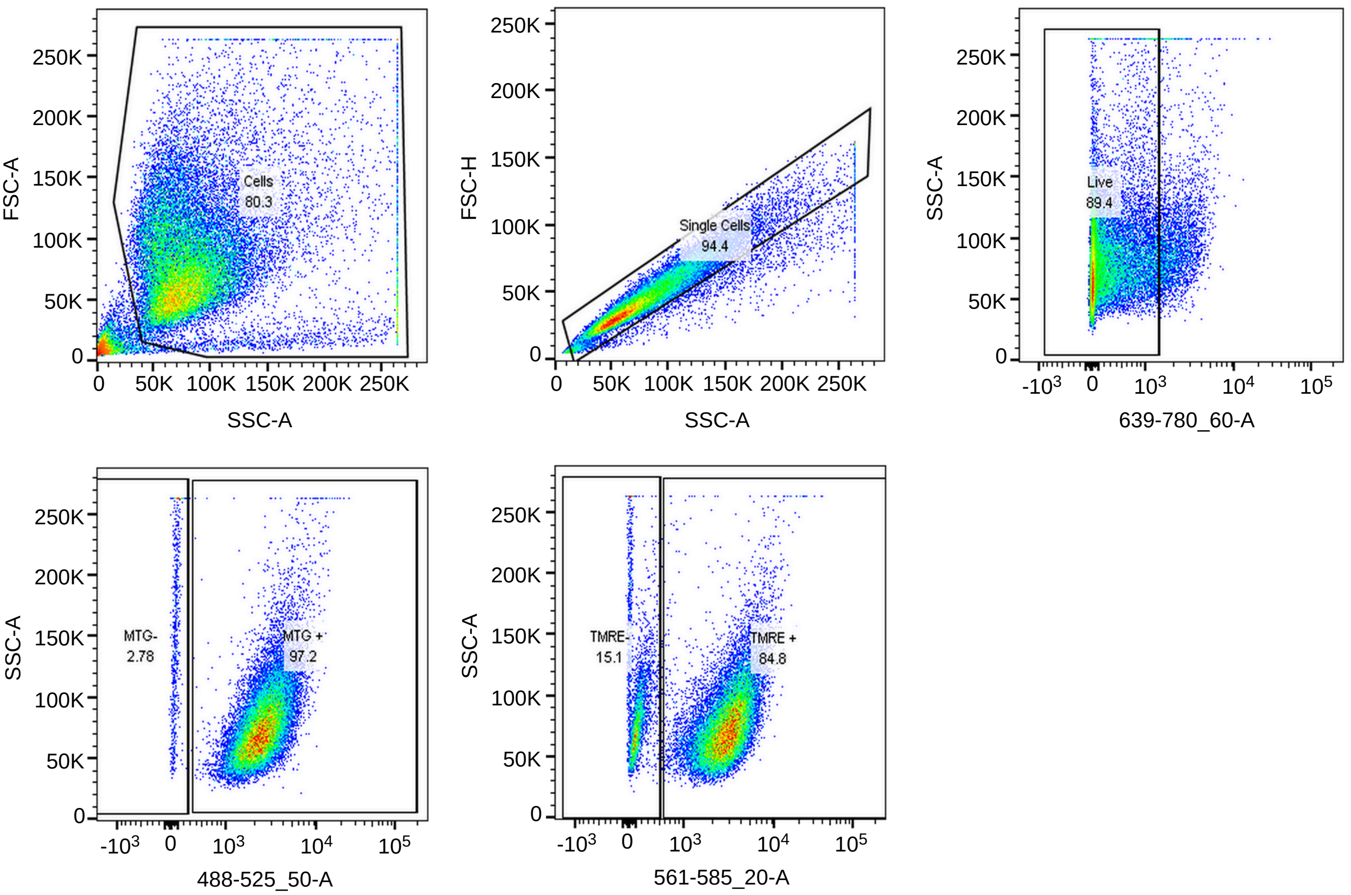

Figure S6. Gating strategy for mitochondrial membrane potential

Gating strategy for experiments shown in Fig. 5C-D and Fig. S3 C-D.

MDAMB231 mitomkeima gating strategy

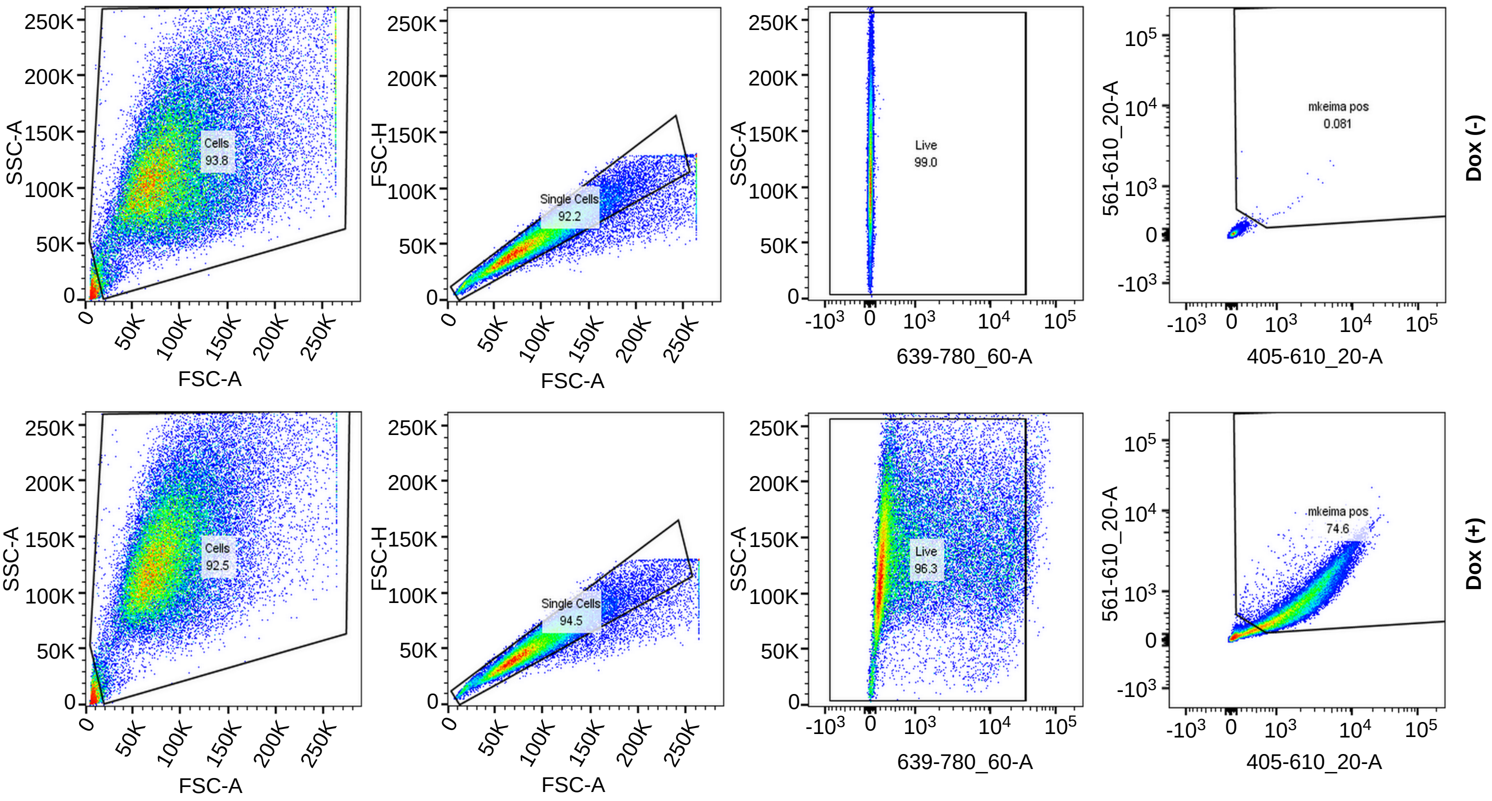

MDAMB231 LDHBmkeima gating strategy

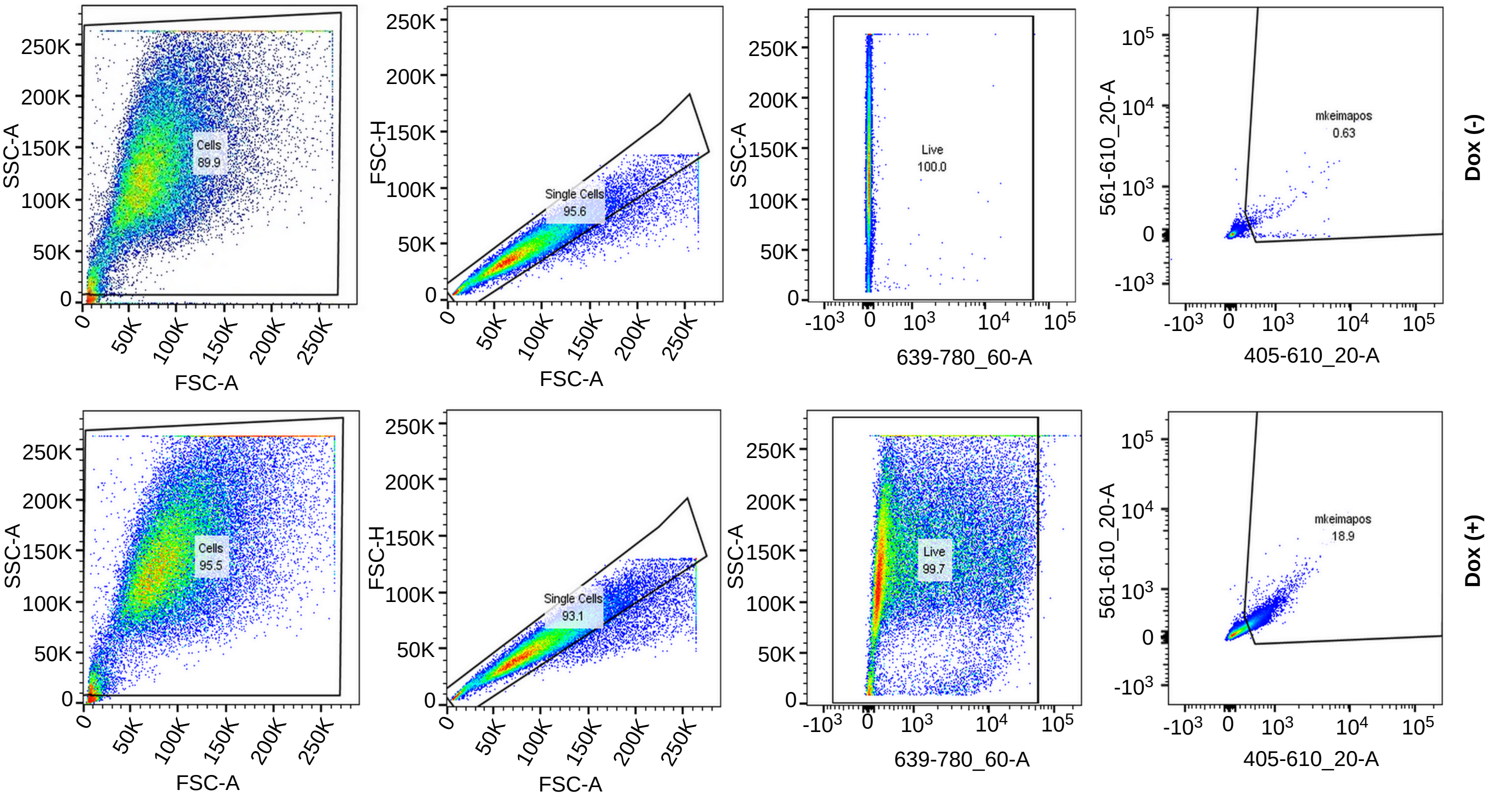

Figure S7. Gating strategy for mitophagy flux determination

Gating strategy for experiments shown in Fig. 5G-I.

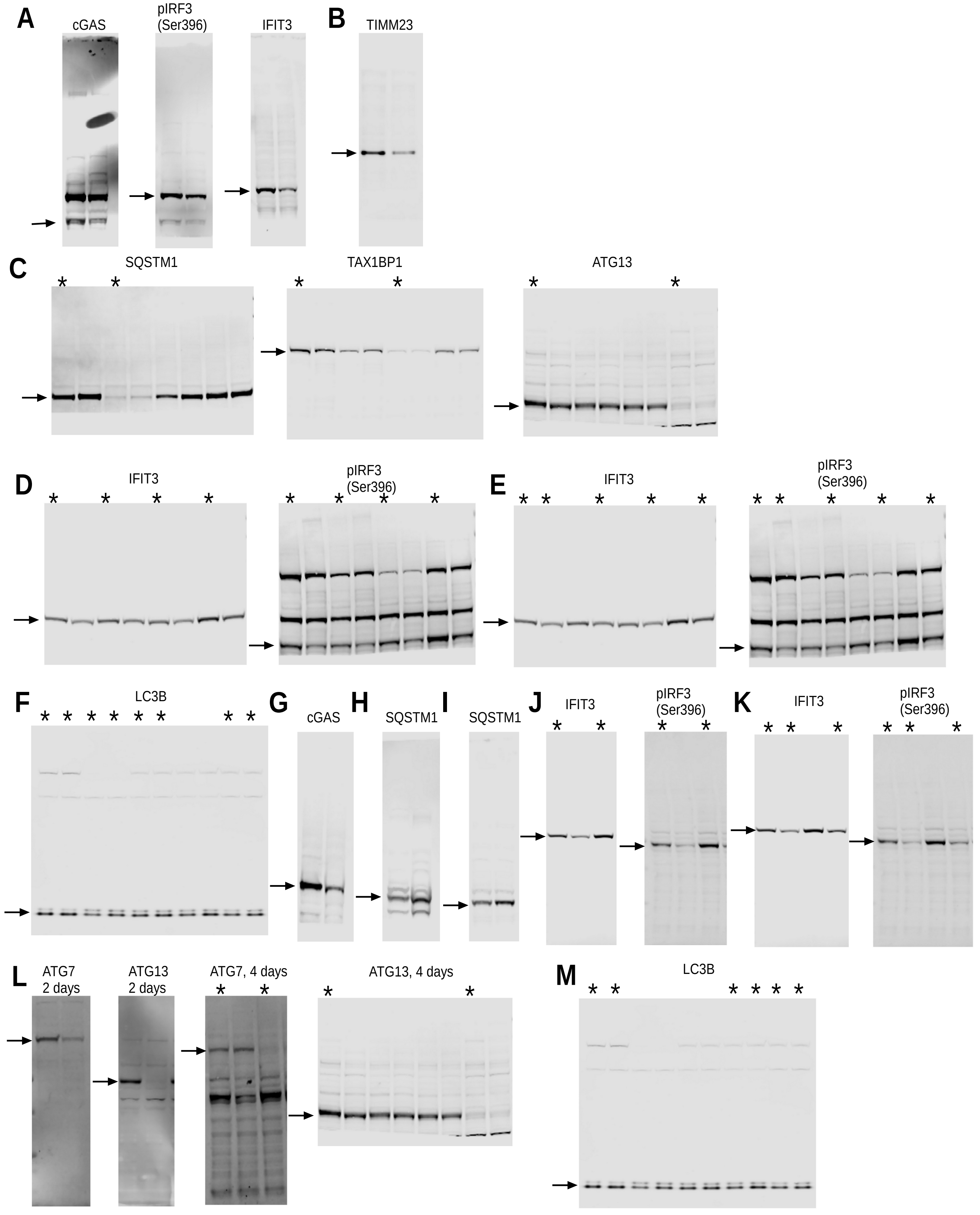

A
cGAS
pIRF3 (Ser396)
IFIT3
B
TIMM23
C
SQSTM1
TAX1BP1
ATG13
D
IFIT3
pIRF3 (Ser396)
E
IFIT3
pIRF3 (Ser396)
F
LC3B
G
cGAS
H
SQSTM1
I
SQSTM1
J
IFIT3
pIRF3 (Ser396)
K
IFIT3
pIRF3 (Ser396)
L
ATG7 2 days
ATG13 2 days
ATG7, 4 days
ATG13, 4 days
M
LC3B

Figure S8. Uncropped western blot images

Uncropped images of western blots presented as cropped versions in Fig 2E (A), Fig 5F (B), Fig 6A (C), Fig 6C (D), Fig 6F (E), Fig 6I (F) Fig S2A (G), Fig S2C (H), Fig S2D (I), Fig S4A (J), Fig S4C (K), Fig S4E (L), and Figure S4G (M). * Indicate lanes included in the cropped versions.
