# Supplementary material for: Elucidating the power of arginine restriction: taming type I interferon response in breast cancer via selective autophagy
Source: Cell Commun Signal. 2024 Oct 8;22:481. doi: 10.1186/s12964-024-01858-6 (PMC11462705; doi:10.1186/s12964-024-01858-6)
Supplement: Supplementary file 2 — Additional file 2. Supplementary methods. [file 12964_2024_1858_MOESM2_ESM.pdf]

SUPPLEMENTARY METHODS

Cell lines and cell culture

66cl4 cells from the 4T1 model were obtained from Barbara Ann Karmanos Cancer Institute, Detroit, MI, USA. The human breast cancer cell line characterized as invasive (MDAMB231) (1, 2) was kindly provided by Dr. Kaisa Lehti (Norwegian University of Science and Technology, NTNU). All cell lines were cultured in Dulbecco's Modified Eagle Medium (DMEM) (Lonza, BE12-604F) supplemented with 10% fetal calf serum (Gibco, 10272-106), 2 mM L-Glutamine (Lonza, BE17-605E), and 50 units per ml/50 µg per ml of Penicillin-Streptomycin (Gibco, 15070-063). Cells were incubated at 37°C with 5% CO_2.

Mice experiments

Eight- to twelve-week-old female BALB/cJ mice were obtained from Janvier Labs, France. The tumors were initiated and resected and processed as in (3). In brief, the mice were injected with 1 x 10^6 viable 66cl4 or 67NR cancer cells into the mammary fat pad. The mice were sacrificed 3 weeks after injection for 67NR tumors and after 4 weeks for 66cl4 tumors. At this point, tumor size was less than 1.5 cm, in accordance with the ethical approval. After resection, the tumors were snap frozen in liquid nitrogen and stored at -80°C. 67NR (N=6) and 66cl4 (N=6) tumors were randomly selected for mass spectrometry analysis. Blinding was not used for any step of the experimental process.

Mass spectrometry analysis for tumor sections

Small pieces of 67NR (N=6) and 66cl4 (N=6) tumors were thawed briefly in lysis buffer followed by homogenization using 1.4 mm ceramic beads (Precellys, 03961-1-103) in reinforced tubes (KT03961-1-403.2) for 4 cycles á 40 sec homogenization, 2 min break. Lysis buffer: 8 M urea (Merck Millipore, #1084870500) with 4 % CHAPS, 100 mM DTT, (Sigma, #646563), 1x Complete® protease inhibitor (Roche, #1187350001) and 2x phosphatase inhibitor cocktail II (Sigma, #P5726) and III (Sigma, #P0044). The homogenized tissue samples were shaken and centrifuged (15 000 g, 20 min, 4°C). Protein concentration was measured at 595 nm using BioRad protein assay dye reagent (Bio-Rad, #500-0006). MS analyses of the samples were performed as described for arginine starved cells. Due to low protein yield in one of the 67NR samples, this sample was withdrawn from any further analysis.

Arginine starvation

SILAC DMEM lacking L-arginine and L-lysine (Thermo Fisher, 88364) was supplemented with 10% dialyzed fetal bovine serum (Gibco, 26400044), 1x non-essential amino acids (Gibco, 11140035) and L-lysine (146.2 mg/L, Sigma-Aldrich, L8662). A varying concentration of L-arginine (Sigma-Aldrich, A6969) was added to prepare full medium (400 µM arginine: 84 mg/L) and medium for partial or complete arginine starvation (40 µM: 84 mg/L, 4 µM: 8.4 mg/L and 0 µM: 0 mg/L). The cells were washed twice with Dulbecco's Phosphate Buffered Saline (DPBS) (Sigma-Aldrich, D8537) before changing the growth medium to SILAC medium with various arginine concentrations.

Mass spectrometry analyses for arginine starved cells

The cells grown in full medium and medium with various arginine concentrations were scraped and lysed in 8 M urea lysis buffer (8 M urea, 4% CHAPS (w/v), 100 mM DTT, 1x Complete® protease inhibitor (Roche, 1187350001), 2x phosphatase inhibitor cocktail II (Sigma-Aldrich, P5726) and III (Sigma-Aldrich, P0044)). The lysates were shaken for 15 min (4°C) before centrifugation (15 000 g, 20 min, 4°C). Protein concentration was measured at 595 nm using BioRad protein assay dye reagent (Bio-Rad, 500-0006). 15 µg of each sample was added to 130 µl 100 mM ammonium bicarbonate, and proteins were reduced and alkylated using 12 mM DTT (30 min, 55°C) followed by 36 mM iodoacetamide (30 min, room temperature (RT) and dark). Proteins were digested with 250 ng trypsin (37°C, overnight) and further acidified in acetic acid (0.5%) and desalted using Oasis HLB C18 solid phase extraction according to manufacturer's instructions. Peptides were eluted from C18, dried in speedvac and dissolved in 18 µl 0.1% formic acid. LC-MS/MS was performed on a timsTOF Pro (Bruker Daltonics) connected to a nanoElute HPLC (Bruker Daltonics). Peptides were separated using a Bruker15 (75 µm*15 cm) column with running buffers A (0.1% formic acid) and B (0.1% formic acid in acetonitrile) with a gradient from 0%B to 37%B for 100 min. The timsTof instrument was operated in the DDA PASEF mode with 10 PASEF scans per acquisition cycle and accumulation and ramp times of 100 millisecond each. The 'target value' was set to 20 000, the dynamic exclusion activated and set to 0.4 min and the quadrupole isolation width was set to 2 Th for m/z < 700 and 3 Th for m/z > 800.

Proteomics data analysis and bioinformatics analysis

The search parameters were set as follows: enzyme specified as trypsin with maximum two missed cleavages allowed; variable modifications as deamidation of asparagine/glutamine,

oxidation of methionine and protein N-terminal acetylation; precursor and fragment mass tolerance were set to 20 parts per million. The parameters were imported in MaxQuant which uses m/z and retention time values to align each run against each other sample with a minute window match-between-run function and 20 min overall sliding window using a clustering-based technique. These were further queried against the mouse proteome along with isoforms downloaded from Uniprot (4) in 2021 including MaxQuant's internal contaminants database using Andromeda built into MaxQuant. Both protein and peptide identifications false discovery rate were set to 1%, thus only unique peptides with high confidence were used for final protein group identification. Integration of the area under the peak curve was used to extract peak abundances. Each protein group abundance was normalized by the total abundance of all identified peptides for each run and for each protein by calculated median summing of all unique and razor peptide-ion abundances for each protein using label-free quantification (LFQ) algorithm (5) with minimum peptides ≥ 1. A log2-transformation was applied to the LFQ values for all samples. The transformed LFQ values were plotted using correlation heatmap to remove potential outliers. The rest of the values for each condition were subjected to two-sided student's t-tests (6) as implemented in R (7) to check the consistency of change. The amount of change was estimated by subtracting the median of these values representing each group (log2 median change). Directionality of the change is encoded within the sign of log2 median change whereby a decreased expression of the respective protein group is reflected by a negative sign and increased expression by a positive sign. Further, to estimate the false-discovery rate (FDR), the t-test p-values were corrected using the Benjamini-Hochberg procedure (8). Differentially expressed (DE) protein groups were identified at an FDR<0.1 and absolute log2 median change >1. The DE proteins quantified only in one group were checked if their coefficient-of-variation of log2 median change was within 5%. The Uniprot accession IDs of these DE proteins were mapped to a volcano-plot using R package ggplot2 (9). Principal component analysis was performed using prcomp function (v4.2.1) in R studio and the plots were generated using ggplot. Volcano plots represented in the figures were drawn using the EnhancedVolcano R package (v 1.0.1), and the cut off was set to log2 median change ranging from ±0.5-±1.0 and the corrected t-test p-value to < 0.05. Log2 median change is represented as log2 fold change (abbreviated log2FC) throughout the article. Heatmaps of expression data from (10) were plotted after log10 transformation of the expression values using pheatmap package (v1.0.12). Functional enrichment analysis of all significantly upregulated and downregulated proteins was performed to identify the common biological functions. Gene ontology (GO) functional enrichment analyses for biological processes (BP) and cellular

components (CC) for DE proteins were conducted using the bioconductor package clusterProfiler (11) from different groups applicable. The plots were plotted using dot plot function (v0.3-1). The colour of the dots indicate the adjusted p-value, where red dots represent the most enriched categories; the 'count' indicates the number of proteins enriched in the GO term; the 'GeneRatio' indicates the proportion of proteins overlapping between lists of differentially expressed proteins and the proteins in GO (BP). The mass spectrometry proteomics data have been deposited to the ProteomeXchange Consortium via the PRIDE (12) partner repository with the dataset identifier PXD037288. All other data in this article can be requested.

Quantitative real-time PCR

High-Capacity cDNA Reverse Transcription Kit (Invitrogen, 4368814) was used for the synthesis of cDNA from 500 ng total RNA. Quantitative real-time PCR (RT-PCR) was performed in 20 µl reactions containing 10 µl of 2X Perfecta SYBR Green PCR master mix (QuantaBio, 733-1386), 2 µl 10X QuantiTect Primer Assay and 8 µl of the sample containing 4 ng of cDNA. RT-PCR was performed on the StepOne plus system (Applied Biosystems) using the following cycling conditions: 95ºC for 15 min, 40 cycles of 94°C for 15 sec, 55°C for 30 sec and 72°C for 30 sec. The $2^{(-\Delta\Delta CT)}$ method was used to calculate relative gene expression levels. Transcripts were normalized to *Actb*.

Primers

QuantiTect Primer Assays were purchased from Qiagen: Mm_*Actb*_2_SG (QT01136772), Mm_*Sqstm1*_1_SG (QT00127855), Mm_T*ax1bp1*_1_SG (QT01067304), Mm_*Bnip3*_1_SG (QT02520126) and Mm_*Bnip3l*_2_SG (QT00120953).

Immunoblotting

Invitrogen 4-12% NuPAGE Bis-Tris protein gels were loaded with equal amounts of proteins (40 µg) for each sample, and blotting was done using iBlot dry blotting system and nitrocellulose membranes. Membranes were blocked in Intercept (TBS) blocking buffer (Li-Cor, 97-60001) mixed 1:1 with TBS (137 mM NaCl, 20 mM Tris-HCl, pH 7.6) containing 0.1% Tween 20 (TBST) for 1 h at RT, and then incubated at 4ºC overnight with primary antibodies as listed under the "Antibodies" section below. After washing in Intercept blocking buffer/TBST-mix, near-infrared fluorescent secondary antibodies were added to detect the proteins of interest (Li-Cor; IRDye 800CW and IRDye 680CW diluted 1:10 000 and 1:20 000,

respectively, in Intercept blocking buffer/TBS-mix 1:1 (no Tween)) and membranes were incubated for 1 h at RT. Membranes were washed (Intercept blocking buffer/TBS-mix, 1:1) and dried before being scanned and analyzed using an Odyssey CLx Infrared Imaging System and Image Studio v3.1 and v5.2 (Li-Cor). For normalization we used Revert 700 total protein stain according to the manufacturer's instructions (Li-Cor, 926-11011).

Immunofluorescence

MDAMB231 cells were grown on high precision cover glass (thickness 0.17 ± 0.005mm; Marienfeld) in 6 well culture plates (100 000 cells/well) to 50-60% confluence. The cells were given SILAC medium with 400 µM or 0 µM arginine for 24 h and 48 h before fixation (details regarding medium in "Arginine starvation"). The cells were fixed with ice cold methanol (10-15 min at -20 ºC) and permeabilized with 0.05% saponin in DPBS for 5 min at room temperature (RT). The cells were incubated with primary antibodies for 1–2 h at RT and washed three times in DPBS with 0.05% saponin before staining with secondary antibodies for 30 min-1 h at RT. Both primary and secondary antibodies were diluted in DPBS containing 0.05% saponin. After antibody staining, and subsequent washing with DPBS with 0.05% saponin, the coverslips were mounted on microscope slides (Menzel–Glaser) with Mowiol (Sigma Aldrich, 81381) containing 10 µg/ml Hoechst 33342 (Invitrogen, H3570) and kept in the dark and at 4ºC until they were imaged. The cells were imaged on a Nikon ECLIPSE Ti2-E inverted microscope (Nikon Corp, Japan) equipped with a CSU-W1 dual spinning disc (50 µm pinholes & 50 µm pinholes with microlenses allowing super resolution SoRa imaging) confocal unit (Yokogawa Electric Corp, Japan), a Prime BSI sCMOS camera (Teledyne Photometrics, AZ, US), a laser unit with 405/488/561/638nm lasers (120/100/100/100mW), and BrightLine single-band bandpass filters (447/60nm, 525/50nm, 600/52nm, 708/75nm) for quantifications. For scoring of micronuclei, regions of interest were randomly selected and Z-stacks with sectioning of 0.6 µm were collected with a 40X Plan Apo λ objective (NA 0,95, Air). The total number of cells was assessed by segmentation of nuclei using Nikon NIS-Elements and then the fraction of micronucleated cells was scored along with the number of micronuclei per micronucleated cell and the number of cGAS positive micronuclei per micronucleated cell. Around 300-500 cells were counted in each of three independent experiments. The figure displays montage of representative images that were processed using ImageJ/FIJI. The montage was created using the "Make Montage" tool in ImageJ/FIJI, combining individual images into a single composite.

For scoring of micronuclei associated with autophagy markers SQSTM1 and LC3B, micronuclei of cells fixed in methanol and immunolabelled for cGAS, SQSTM1 and LC3B were imaged with a SoRa super resolution mode and by using a CFI Plan Apo λ 100x (NA 1.54 Oil) objective. The mean intensity of SQSTM1 and LC3B at cGAS positive micronuclei was measured by ImageJ. First, micronuclei were segmented using the cGAS channel by Otsu thresholding. The ImageJ function "analyze particles" was then used to define regions of interest (ROIs) and the mean intensity were measured in the channels of SQSTM1 and LC3B. The mean intensity of both channels was also measured in background areas and the values were subtracted to the values measured in the micronuclei ROIs. For plotting of the data, each experiment was normalized to the mean of the control. The number of LC3 puncta at cGAS-positive micronuclei was measured by a Nikon NIS-Elements AR software. Micronuclei were segmented using the Hoechst channel first, then using the cGAS channel. Within the segmented ROIs, "shading correction" was applied to the LC3 channel to subtract background signal. LC3 spots localizing inside and intersecting the ROI were then detected by the function "having". For plotting of the data, each experiment was normalized to the mean of the control. Representative images collected by super resolution imaging were deconvolved using NIS-Elements AR software and processed in ImageJ/FIJI. Figure panels show the z-projection of 3 planes.

Antibodies

The following antibodies were used for western blot (WB) or immunofluorescence (IF): anti-pIRF3 (Cell Signaling Technology, 29047, 1:1000 for WB), anti-IFIT3 (E-10) (Santa Cruz Biotechnology, sc-393396; 1:500 for WB), anti-cGAS (D1D3G, human specific, Cell Signaling Technology, 15102; 1:100 for IF and 1:1000 for WB), anti-cGAS (D3080, mouse specific, Cell Signaling Technology, 31659; 1:1000 for WB), anti-Lamin A (abcam, ab8980; 1:100 for IF), anti-SQSTM1 (Progen, GP62-C; 1:200 for IF and 1:1000 for WB), anti-LC3B (Nanotools, 0231-100/LC3-5F10; 1:100 for IF), anti-LC3B (Cell signaling Technology, 3868, 1:1000 for WB), anti-ATG7 (Cell signaling Technology, 8558, 1:1000 for WB), anti-TIMM23 (Santa Cruz Biotechnology, sc-514463; 1:500 for WB), anti-ATG13 (Sigma-Aldrich, SAB-4200100; 1:1000 for WB), anti-TAX1BP1 (Cell Signaling Technology, 5105; 1 :1000 for WB). Fluorescent secondary antibodies (Li-Cor; IRDye 800CW and IRDye 680CW diluted 1:10 000 and 1:20 000, respectively) were used in WB. The following secondary antibodies

were used for IF: donkey anti-mouse Alexa Fluor 568 (Molecular Probes, A10037; 1:500) and donkey anti-rabbit Alexa Fluor 488 (Jackson, 711605152; 1:500).

LDH sequestration assay for evaluation of bulk autophagy

66cl4 cells (400 000 cells per well) and MDAMB231 (140 000 cells per well) were seeded in 6 well plates (three independent experiments in triplicate wells) and grown to 60-70% confluency. The cells were grown either in SILAC medium with 400 µM arginine (full medium), medium without arginine (-R) or medium without any amino acids (HBSS) for 24 h, or in full medium with Torin (50 nM, overnight). The cells were treated with BafA1 (100 nM, last 3 h before harvesting), except for the full medium controls. Details regarding medium is shown in the section about "Arginine starvation". Cells were detached by trypsin-EDTA (0.25%) and collected in full medium. After centrifugation at 400 × g for 5 min at 4°C, the supernatant was aspirated, and cells were resuspended in 400 µl of isotonic sucrose (10%). To selectively disrupt the plasma membrane, cells were subjected to an electric pulse (2000 V and 1.2 microfarads in a 1 × 1 × 5-cm electrode chamber) with a homemade apparatus, and subsequently mixed with 400 µl of phosphate-buffered sucrose (100 mM sodium monophosphate, 2 mM DTT, 2 mM EDTA, and 1.75% sucrose, pH 7.5) to a total volume of ~ 750 µl. 150 µl of cell disruptate was removed for total LDH measurements ("LDH Total") and was stored overnight at −80°C. 550 µl of cell disruptate was resuspended in 900 µl of resuspension buffer (50 mM sodium monophosphate, 1 mM EDTA, 1 mM DTT) supplemented with 0.5% BSA and 0.01% Tween 20. Cell corpses, containing autophagic vacuoles, were sedimented by centrifugation at 18 000 ×g for 45 min at 4°C. Supernatant was aspirated, and the pellet ("LDH Sediment") was stored overnight at -80°C. The following day, both LDH Total and LDH Sediment were diluted in resuspension buffer supplemented with Triton X-405 (Sigma-Aldrich, X-405) to a final concentration of 1%. After centrifugation (18 000 × g for 10 min at 4°C), the enzymatic activity of LDH in LDH Total and LDH Sediment was measured as described previously (13) using homemade reagents, mixing 4 volumes of 65 mM imidazole (pH 7.5), 0.75 mM pyruvate with one volume of 65 mM imidazol (pH 7.5), 1.8 mM NADH. LDH sequestration activity was calculated as percentage of sedimentable LDH in experimentally treated cells minus percentage of sedimentable LDH in untreated cells (background), divided by the incubation time with BafA1. For a detailed description of the protocol, see Luhr et al. (13).

Metabolic analyses of glycolytic and mitochondrial function using Seahorse XF96 Analyzer

66cl4 cells (13 000 cells/well) were seeded in regular growth medium (80 µl/well) in XF96 cell culture microplates (part of the Seahorse XFe96 FluxPak, Agilent, 102416-100) that had been pre-treated with 0.01% poly-l-lysine (Sigma-Aldrich, P4707) for ~1 h at RT and washed once in sterile water before adding the cells. The following day, the cells were 60-70% confluent, they were gently washed in warm DPBS (250 µl/well) and grown for 24 h in SILAC medium (100 µl/well) with 400 µM, 40 µM, 4 µM or 0 µM arginine (details on medium composition were as described in the section "Arginine starvation"). We performed both a glycolysis stress test and a mito stress test on the same plate, dedicating at least 11 wells to each condition. Following incubation in medium with varying arginine concentrations, the cells were washed twice in medium designed either for glycolysis or mito stress test (100 µl/well) and then incubated (180 µl medium/well) in a CO_2-free incubator for ~1 hour to adapt to the CO_2-free condition and the new medium. For the two assay variants, we used serum-free Seahorse XF Base Medium (Agilent, 102353-100) supplemented with either 6 mM glutamine (glycolysis stress test) or a combination of 10 mM glucose, 10 mM pyruvate and 6 mM glutamine (mito stress test), and for both tests, the pH of the supplemented medium was adjusted to 7.4 at 37°C. Before performing the metabolic measurements on the treated cells, a cartridge (part of the Seahorse XFe96 FluxPak) with sensors measuring changes in extracellular pH and partial oxygen pressure had been rehydrated, loaded with the agents to be injected during the measurements and calibrated according to the manufacturer's instructions.

For the glycolysis stress test, four measurements of the basal extracellular rate (ECAR) were performed before the sequential injections of a saturating concentration of glucose (10 mM), oligomycin (1 µM) and finally 2-deoxy-glucose (50 mM). For the mito stress test,four measurements of the rate (OCR) were performed before the sequential injections of oligomycin (1 µM), carbonylcyanide p-trifluoromethoxyphenylhydrazone (FCCP; 1.5 µM) and the combination of antimycin A (1 µM) and rotenone (1 µM). We used a cycle of 2 min mixing + 4 min measuring for the initial, basal measurements, and then 7 min mix + 4 min measuring for all measurements performed after injections. This had been optimized to fit the cell line in question (66cl4), with both the glyco stress test and the mito stress test being performed simultaneously on the same plate.

After the measurements, the medium in all wells was removed and the plates were sealed and kept at -80°C for up to 4 weeks. We then lysed the cells and stained their DNA using the CyQUANT™ Cell Proliferation Assay (Invitrogen, C7026), following the manufacturer's

instructions. The raw data from the fluorescence measurements (Ex485, Em535) were used for normalization of the ECAR and OCR data, thus correcting for variance in cell numbers. Seahorse Wave (version 2.6.1) Desktop software was used for initial data analysis, while GraphPad Prism version 9 was used for statistical analyses and generation of curves and graphs.

Analysis of mitochondrial membrane potential

66cl4 cells (28 500 cells per well) and MDAMB231 (28 000 cells per well) were seeded in 24 well plates (three independent experiments in triplicate wells) and grown to 60-70% confluency. The cells were then grown in SILAC medium with varying arginine concentrations for 24 h (66cl4 cells) or 48 h (MDAMB231 cells) (details on medium composition were as described in the section "Arginine starvation"). The cells were stained with 200 nM tetramethylrhodamine ethyl ester perchlorate (TMRE) (Invitrogen, T669) and 300 nM Mitotracker Green (MTG) (Invitrogen, M46750) for 30 min at 37°C. The cells were harvested by trypsinization, and then stained with Fixable viability stain 780 (FVD) (Invitrogen, 65-0865-14; 1:1000 in DPBS) for 30 min on ice. The cells were washed twice and resuspended in FACS buffer (DPBS with 2% FCS and 0.2 mM EDTA) and ran on a BD LSR II flow cytometer recording 50 000 events per well. Fcs files were analyzed in the FlowJo10.2 software as follows. Doublets and dead cells were excluded, followed by gating for TMRE and MTG positive cells (Fig. S6). Mitochondrial abundance and mitochondrial membrane potential (MMP) were assessed by determination of the median fluorescence intensity (MFI) of MTG and TMRE, respectively. The MFI of TMRE was normalized to the MFI of MTG to determine the mitochondrial activity of the mitochondria present within each cell population.

Generation of stable cell lines expressing inducible mito-mKeima or LDHB-mKeima

MDAMB231 cells were grown in 6 well cell culture plates until 70% confluency. The lentiviral constructs were a kind gift from Dr. Lisa Frankel (Danish Cancer Society Research Center). Dr. Maria L. Torgersen (Oslo University Hospital) used these constructs to generate the viruses as in(14) and kindly shared these viruses with us. The cells were incubated with or without 500 µl of the respective virus, in addition to 8 µg/ml polybrene. The cells were washed with DPBS and given fresh culture medium 19 h after infection. The cells were split 24-48 h after starting the infection, and stably transduced cells were selected by maintaining the cells in 2 µg/ml puromycin (Sigma-Aldrich, P8833) over five passages. Thereafter, the cells were cultured without puromycin. To turn on the expression of the mKeima variants, the cells were treated

with 500 ng/ml doxycycline (Sigma-Aldrich, D9891) and the activity of the constructs were validated by confocal microscopy and flow cytometry as in (14).

Assessing autophagic flux by flow cytometry

MDAMB231-mt-mKeima and MDAMB231-LDHB-mKeima cells were seeded in 6 well plates (140 000 cells per well) with regular DMEM containing 500 ng/ml doxycycline and grown for 2 days to 60-70% confluency. The cells were then grown in SILAC-medium with 400 µM or 0 µM arginine for 48 h (details on medium composition were as described in the section "Arginine starvation"). The cells were washed twice with DPBS, trypsinized, the trypsin was inactivated using DPBS with 20% dialyzed FBS (Gibco, 26400044), and cells were sedimented by centrifugation at 400 x g for 5 min at 4°C. The cell pellets were resuspended in 150-400 µl FACS buffer (DPBS with 2% FCS and 0.2 mM EDTA) and stained with Fixable Blue Dead Cell Stain UV (Invitrogen, L23105; 1:1000 in DPBS) for 30 min on ice. The cells were washed twice, resuspended in FACS buffer, and analyzed on a BD LSR II flow cytometer in biological and technical triplicates recording 200 000 events per sample. mKeima was excited in each cell by 407 nm and 561 nm lasers, and a 610/20 bandpass filter and 600 nm long pass dichroic filter were used to acquire the emission (14). The fcs files were analyzed in the FlowJo10.2 software. Cell gates were set after the exclusion of doublets and dead cells, followed by gating of mKeima-positive cells (Fig. S7). Designated live, single cells positive for mKeima were plotted for the fluorescent signals obtained from excitation with the 407 nm laser versus the 561 nm laser. For downstream ratiometric analyses, the fluorescence intensity obtained for each cell from each sample with the 561 nm laser was divided by that obtained with the 407 nm laser, using the "Derive Parameters" function in FlowJo, and the ratio values were plotted as histograms for each treatment condition, as in (14). The median ratio value from each sample was normalized to that obtained in the sample from control cells, where the median ratio value from control cells was arbitrarily set to 100.

Knocking down autophagy-associated genes by siRNAs

Cells were seeded and transfected with siRNAs simultaneously. The siRNAs (final concentration 20 nM) and the DharmaFECT 1 transfection reagent (Dharmacon, T-2001-03; 6 µl/well in 6 well plates) were all diluted in Optimem (Gibco, 11058021) in accordance with the manufacturer's recommendations for the transfection reagent, and mixed with the cell suspension (450 000 cells/well) in a total volume of 2.5 ml. Following 24 h, the medium with transfection agent was replaced with regular growth medium. After 24 h in regular medium,

the cells were given SILAC medium with 400 µM or 0 µM arginine for 48 h before immunoblot analysis (details on medium composition were as described in the section "Arginine starvation"). When assessing the ability of siRNAs to inhibit autophagy the cells were transfected as described above. The medium was exchanged to regular growth medium, and the cells were grown for 24 h, with Baf A1 treatment (100nM) for the last 6 h. The following siRNA oligonucleotides were used: non-targeting siRNA (Dharmacon, D-001210-01-20); SQSTM1 siRNA (Dharmacon, L-010230-00-0020); TAX1BP1 siRNA (Dharmacon, L-016892-00-0005), ATG7 siRNA (Dharmacon, L-020112-00-0005) and ATG13 siRNA (Ambion Life Technologies, siRNA ID s18879, 4392421).

REFERENCES

1. von Nandelstadh P, Gucciardo E, Lohi J, Li R, Sugiyama N, Carpen O, et al. Actin-associated protein palladin promotes tumor cell invasion by linking extracellular matrix degradation to cell cytoskeleton. Mol Biol Cell. 2014;25(17):2556-70.
2. Sugiyama N, Gucciardo E, Tatti O, Varjosalo M, Hyytiainen M, Gstaiger M, et al. EphA2 cleavage by MT1-MMP triggers single cancer cell invasion via homotypic cell repulsion. J Cell Biol. 2013;201(3):467-84.
3. Lamsal A, Andersen SB, Johansson I, Vietri M, Bokil AA, Kurganovs NJ, et al. Opposite and dynamic regulation of the interferon response in metastatic and non-metastatic breast cancer. Cell Commun Signal. 2023;21(1):50.
4. UniProt. UniProtKB - H3BJL3 (H3BJL3_MOUSE): Uniprot; 2021 [Available from: (https://www.uniprot.org/proteomes/UP000000589.
5. Cox J, Hein MY, Luber CA, Paron I, Nagaraj N, Mann M. Accurate proteome-wide label-free quantification by delayed normalization and maximal peptide ratio extraction, termed MaxLFQ. Mol Cell Proteomics. 2014;13(9):2513-26.
6. Student. The Probable Error of a Mean. Biometrika. 1908;6(1):1-25.
7. project R. The R Project for Statistical Computing [Available from: https://www.r-project.org/.
8. Hochberg YBaY. Controlling the False Discovery Rate: A Practical and Powerful Approach to Multiple Testing: Wiley for the Royal Statistical Society,Journal of the Royal Statistical Society. Series B (Methodological); 1995 [Available from: https://www.jstor.org/stable/2346101.
9. Wickham H. ggplot2: Elegant Graphics for Data Analysis: SpringerLink; 2009 [Available from: https://www.springer.com/gp/book/9780387981413.
10. Cheng CT, Qi Y, Wang YC, Chi KK, Chung Y, Ouyang C, et al. Arginine starvation kills tumor cells through aspartate exhaustion and mitochondrial dysfunction. Commun Biol. 2018;1:178.
11. Yu G, Wang LG, Han Y, He QY. clusterProfiler: an R package for comparing biological themes among gene clusters. OMICS. 2012;16(5):284-7.
12. Perez-Riverol Y, Bai J, Bandla C, Garcia-Seisdedos D, Hewapathirana S, Kamatchinathan S, et al. The PRIDE database resources in 2022: a hub for mass spectrometry-based proteomics evidences. Nucleic Acids Res. 2022;50(D1):D543-D52.
13. Luhr M, Szalai P, Engedal N. The Lactate Dehydrogenase Sequestration Assay - A Simple and Reliable Method to Determine Bulk Autophagic Sequestration Activity in Mammalian Cells. J Vis Exp. 2018(137).
14. Engedal N, Sonstevold T, Beese CJ, Selladurai S, Melcher T, Simensen JE, et al. Measuring Autophagic Cargo Flux with Keima-Based Probes. Methods Mol Biol. 2022;2445:99-115.
